# Impact of Porous Transport Layer Morphology on the Performance of Proton Exchange Membrane Water Electrolyzers with Ultra-Low Iridium Loadings

**DOI:** 10.1021/acsami.6c01544

**Published:** 2026-04-01

**Authors:** Jacob A. Wrubel, Makenzie Parimuha, Sarah Blair, Haoran Yu, Jack Todd Lang, Abigail J. Schmeiser, James L. Young, Elliot Padgett, Iryna V. Zenyuk, Guido Bender

**Affiliations:** † 53405 National Laboratory of the Rockies, Golden, Colorado 80401, United States; ‡ 6146 Oak Ridge National Laboratory, Oak Ridge, Tennessee 37830, United States; § University of California, Irvine, Irvine, California 92697, United States

**Keywords:** hydrogen, PEM electrolysis, durability, porous transport layer, iridium loading, catalyst utilization

## Abstract

Reducing Ir loadings in proton exchange membrane water electrolyzer anodes is critical for lowering capital expenses. Loading reduction could be achieved by improving the Ir activity via doping/alloying and/or the development of advanced microstructures. However, the anode porous transport layer (PTL) is a comparatively simple component whose properties also impact Ir utilization. Therefore, well-designed PTLs may also enable reduced Ir loadings. In this work, we survey eight PTLs from various manufacturers to observe their impact on cell performance at low (0.4 mg_Ir_ cm^–2^) and ultralow (0.1 mg_Ir_ cm^–2^) Ir loadings. The PTLs were characterized by their microstructural properties, including porosity, particle size distribution, and pore size distribution. Electrochemical cell performance was correlated to PTL morphology, and it was found that PTLs with lower porosities and smaller particle and pore radii enabled good performance even at ultralow Ir loadings. 1000-h durability testing indicated that using lower porosity PTLs can significantly improve durability behavior. A runaway voltage phenomenon was observed during durability testing of cells with ultralow Ir loadings, which was caused by increases in both anode and cathode overpotentials. Furthermore, we observed that the beginning of test performance of 0.1 mg_Ir_ cm^–2^ cells correlates to the 1000-h degradation rates of 0.4 mg_Ir_ cm^–2^ cells, suggesting that for the Ir catalyst used in this work, short-term testing at ultralow loadings can be used as an indicator of long-term degradation at higher loadings.

## Graphical abstract

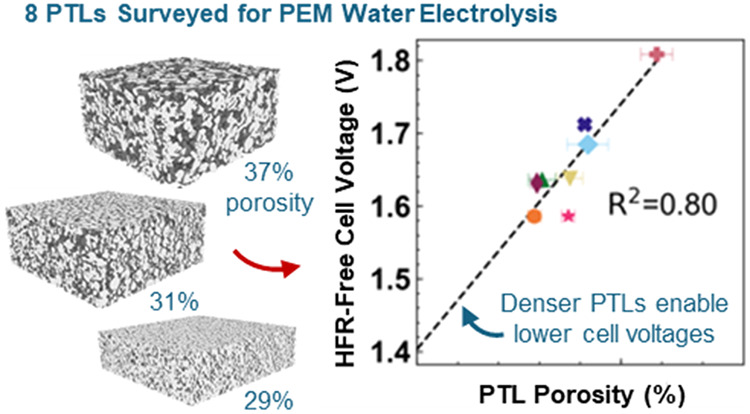

## 1 Introduction

Proton exchange membrane water electrolysis (PEMWE) is a promising technology for hydrogen production, but requires improvements to capital expenditures (CapEx) to achieve cost targets and become competitive with other technologies.^1–4^ While expanding manufacturing to achieve economies of scale will have the biggest impact on CapEx,^1^ advances in membrane electrode assembly (MEA) components are also important. Specifically, increasing the maximum operating current density and reducing the loading of platinum group metals (PGMs) in the MEA can significantly contribute to reduced CapEx. Improving the lifetimes of these components will also be necessary.^4–7^

The anode catalyst layer (ACL) has received much research attention due to the required usage of expensive PGMs, particularly Ir, as catalyst materials.^8^ The high overpotentials associated with the oxygen evolution reaction (OER), coupled with high degradation rates in the harsh environment of the PEMWE anode (low pH, highly oxidizing potentials) typically mandate high loadings of anode catalyst compared to, e.g., those observed in PEM fuel cells.^7,9–11^ Efforts to improve Ir-based anode performance have included morphology regulation, controlling the oxide phase, doping with Ru, and adding conductive supports.^8,12,13^ Porous transport electrode architectures have also demonstrated a capacity to exhibit high Ir utilizations.^14,15^ Nevertheless, the optimum balance across performance, durability, and cost remains to be definitively solved.

The anode porous transport layer (PTL) is responsible for facilitating electron removal from the ACL as electrons are produced in the OER. Recent studies have found that the PTL significantly impacts the Ir utilization in the ACL, concluding that it is the ACL/PTL coupling, not just the ACL properties alone, that dictate anode performance.^16,17^ Due to the low in-plane electrical conductivity of typical ACL architectures (i.e., unsupported Ir), additional overpotentials arise when electrons are forced to travel large distances, e.g., tens of micrometers in the in-plane direction.^18^ While the ACL requires advanced manufacturing techniques to simultaneously disperse mixtures of Ir nanoparticles, ionomer, and solvents, PTLs are thicker, single-phase porous materials with morphological features at the 1–100 μm scale. In other words, the PTL is a less complex component compared to the ACL that can also be leveraged to improve cell performance and reduce CapEx.

Many studies to date have found the morphology of the PTL to be an important driver of cell performance. For example, Schuler et al. reported that denser, smoother surface layers can reduce cell voltages and hydrogen crossover at high current densities.^19^ Duarte et al. reported similar findings, including reduced membrane deformation at elevated cathode pressures with denser PTLs, potentially supporting use of thinner membranes.^20^ Multiple studies have shown that the PTL morphology strongly impacts Ir utilization, with lower PTL porosities and smaller particle sizes leading to increased Ir utilization.^9,18,21^ In PEMWE, Ir utilization refers to the distribution of OER across the Ir surface area, with good utilization leading to uniform OER rates and poor utilization resulting in some areas of the ACL being less active than others. With better utilization comes lower average local OER rates, whereas poor utilization forces some regions to have locally higher OER rates. Locally higher OER rates, and hence overpotentials, can lead to increased Ir dissolution.^21^ Padgett et al. developed a technique for quantifying the Ir utilization *in operando* and found that PTLs with higher porosities resulted in decreased mass activities (and hence decreased Ir utilization) in the cell.^22^

In this work, we quantify the impact of eight different PTLs on the performance of PEMWE cells with both low (0.4 mg_Ir_ cm^–2^) and ultralow (0.1 mg_Ir_ cm^–2^) Ir loadings (as defined in previous works^23^). O*perando* cell performance metrics were correlated to quantitative morphological properties of the PTLs obtained using synchrotron-based computed tomography (CT). Durability testing in the form of 1000-h current holds was performed on a subset of the PTLs, and the resulting degradation rates were also correlated to the PTL morphology. Furthermore, we show that beginning of test (BOT) PEMWE cell performance at ultralow Ir loadings can be correlated to cell durability at low loadings, potentially offering a rapid first order durability assessment method. The results presented here demonstrate that advanced PTL designs can improve Ir utilization, ultimately enabling PEMWE cells with reduced Ir loadings.

## 2 Experimental Section

### 2.1 Materials Preparation

Electrode inks were prepared by mixing catalyst powders, DI water (18 MΩ cm), n-propanol (HPLC-grade OmniSolv), and Nafion ionomer dispersion (D2020, 920EW). For all samples, the anode catalyst material was unsupported IrOx (Alfa Aesar Premion, 99.99%) and the cathode catalyst was Pt/C (TKK TEC10E50E, 46.7 wt % Pt). Inks were first horn sonicated (60s for anodes, 30s for cathodes) and then bath sonicated (60 min for anodes, 30 min for cathodes) prior to spray coating.

Catalyst coated membranes (CCMs) were prepared by spray coating electrode inks onto Nafion 115 (N115) membranes. A Sono-Tek ExactaCoat ultrasonic spray system (25 kHz Accumist nozzle) was used to deposit the inks. During the coating process, the membranes were held in place by a vacuum plate heated to 80 °C. A 5 cm^2^ geometric active area was used for all tests, with a ∼2 mm oversized electrode spray pattern. Ink flow rates of 0.3 and 0.2 mL min^–1^ were used for the anodes and cathodes, respectively. The spray head path speed was 50 mm s^–1^ for both sides. Catalyst loadings were verified using X-ray fluorescence: anodes targeting 0.4 mg_Ir_ cm^–2^ featured average loadings of 0.396 ± 0.008 mg_Ir_ cm^–2^, and anodes targeting 0.1 mg_Ir_ cm^–2^ featured average loadings of 0.130 ± 0.005 mg_Ir_ cm^–2^. All cathodes in this work targeted 0.1 mg_Pt_ cm^–2^ and featured average loadings of 0.113 ± 0.001 mg_Pt_ cm^–2^. CCMs of each loading were all sprayed concurrently in a single batch to improve reproducibility between tests.

As-received PTL materials were first laser cut to 5 cm^2^ (2.236 cm × 2.236 cm) using a Tykma Minilase laser marking system. Once cut, they were etched in Multi-Etch solution at 25 °C for 10–20 min total. Sufficient etching time was determined by measuring the interfacial contact resistances of separate samples before and after etching to observe the time required for the resistances to plateau at a minimum value. PTLs were flipped halfway through to ensure even exposure to solution and then rinsed in DI and sonicated for 30 min to remove all residual etchant. The PTLs were then Pt coated on both sides by DC magnetron sputter deposition in a Denton Desktop Pro system using a 2”-diameter Pt sputtering target (99.99% ACI Alloys, Inc.). After loading the uncoated PTLs, the deposition chamber was evacuated to 5 × 10^–5^ Torr before flowing Ar gas (UHP 99.999% pure) at 50 sccm to establish a deposition pressure of 12–13 mTorr. The sputtering power was 20 W and the sample stage was rotated at 5 rpm. Presputtering at the same conditions was conducted for 2 min before opening the shutter for a deposition time of 30 min, yielding loadings of 0.23 mg_Pt_ cm^–2^ as measured by X-ray fluorescence (XRF) spectroscopy (Fischer XDV-SDD).

### 2.2 Cell Testing

The prepared CCMs were tested using in-house low temperature electrolysis cells featuring triple serpentine flow channels. PTFE gaskets, used to align the anode PTL and cathode gas diffusion layer (GDL), were cut to size with a Silhouette Cameo 3. The anode gasket thicknesses were selected to exactly match the PTLs being tested. The cathode gasket thickness was 0.010″, which was selected to target 20% compression of the GDL (AvCarb MGL280 for all tests). All cells featured a geometric active area of 5 cm^2^. The cells were assembled by tightening the bolts first to 20 in*lb, then 40 in*lb. During testing, water was supplied to the anode side only at a flow rate of 50 mL min^–1^. Both the inlet water temperature and flow field temperature were controlled to 80 °C using inline water heaters, end plate pad heaters, and Watlow EZ zone controllers.

For all experiments, cells were conditioned using the following ∼32 h procedure:

- 1.5 V potentiostatic hold, 5 min
- 1.6 V potentiostatic hold, 5 min
- 1.7 V potentiostatic hold, 5 min
- 1.8 V potentiostatic hold, 10 h
- 11 current-controlled polarization curves featuring 3 min holds at the following current densities: 0.01, 0.015, 0.02, 0.03, 0.04, 0.05, 0.075, 0.1, 0.15, 0.2, 0.3, 0.4, 0.5, 0.75, 1, 1.5, 2, 3, and 4 A cm^–2^
  - Both up (0.01 → 4 A cm^–2^) and down (4.0 → 0.01 A cm^–2^) scans were conducted for each polarization curve

Electrochemical diagnostics were collected using a Gamry Reference 3000 potentiostat/galvanostat and booster. Polarization curves and electrochemical impedance spectroscopy (EIS) spectra were collected at current densities of 0.01, 0.02, 0.05, 0.075, 0.1, 0.2, 0.35, 0.5, 0.75, 1, 1.5, 2, 3, 4, and 5 A cm^–2^. EIS was carried out between 40 kHz and 0.1 Hz with an AC amplitude of 5% of the DC signal, and with 10 points/decade.

### 2.3 Characterization

To prepare cross sections for microscopy analysis, portions of the tested CCMs were embedded in epoxy resin and then cut by diamond-knife ultramicrotomy with a target thickness of ∼75 nm. High-angle annular dark-field scanning transmission electron microscopy (HAADF-STEM) and energy-dispersive X-ray spectrum (EDS) images were recorded using a JEM-2100F analytical electron microscope (JEOL Ltd.) operated at 200 kV. The EDS data were acquired and processed using AZtec (Oxford Instruments) software.

Ex-situ X-ray micro computed tomography (CT) for each PTL sample was performed at beamline 8.3.2 at the Advanced Light Source at Lawrence Berkeley National Laboratory. A white light X-ray beam was used for imaging. A 50 μm LuAG scintillator, a sCMOS PCO Edge camera, and an optical magnification of 10x were used for the image capture. A resolution of 0.65 μm/pixel was achieved with this setup. Each sample was rotated 180 deg while 2625 back projections were captured. At an exposure time of 80 ms per projection and including time for instrument adjustments, the scans took 10 min per sample. The data sets were reconstructed and processed with Tomopy and Gridrec algorithms, as discussed further in previous work.^18,19^ The resulting image stacks consisted of 2160 image slices. These image stacks were converted from 32-bit to 8-bit, cropped, and manually thresholded in ImageJ. PTL sample porosity was calculated from the binarized image stacks using an ImageJ macro. The ImageJ plugin, BoneJ, was used to calculate average pore and particle sizes, as well as pore and particle size distributions for the samples.

### 2.4 Reference Electrode

Reference electrode measurements were obtained for additional information during cell durability testing. The reference electrode is a standalone apparatus that was integrated with our standard PEMWE hardware as described previously.^24,25^ The electrode consists of two end plates compressing Pt-coated MGL280 (AvCarb) and a stainless-steel current collector between silicone gaskets onto a length of N115 extending from the CCM out of the PEMWE hardware. Humidified H_2_ gas was flowed through the apparatus to create a reversible hydrogen electrode (RHE), enabling independent measurement of anode and cathode potentials via a multichannel potentiostat.

## 3 Results and Discussion

### 3.1 Morphology Characterization

Figure 1 shows X-ray CT reconstructions for seven of the eight PTLs studied in this work, with one sample’s image withheld at the manufacturer’s request. The PTLs feature a range of particle types, thicknesses, and morphologies. Table 1 lists the quantitative morphological parameters extracted from the reconstructions in Figure 1. The PTLs are listed in order of decreasing porosity (ranging from ∼60 to 30%) and are referred to using only their sample ID, as the goal of this work is solely to identify structure-properties-performance relationships and not to endorse any particular product, material architecture, or vendor.

**Figure 1:**
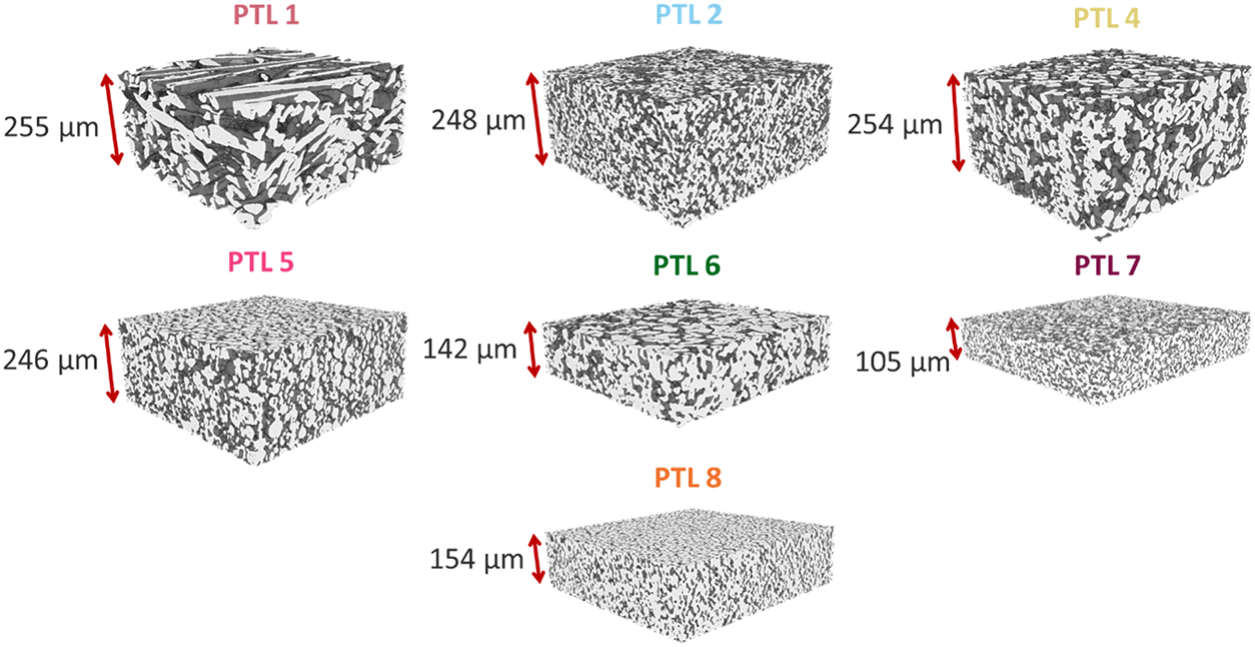
X-ray CT reconstructions of the PTLs studied in this work (PTL 3 not included at manufacturer’s request).

**Table 1:** Morphological Properties of the Eight PTLs Studies in This Work (from X-ray CT); [±] Refers to the Standard Deviation of the Size Distribution

PTL ID	thickness, μm [±]	porosity, % [±]	avg. pore radius, μm [±]	avg. particle radius, μm [±]
1	255 [5.8]	58.8 [3.9]	15.1 [9.1]	13.0 [5.4]
2	248 [6.3]	41.9 [5.0]	4.30 [1.9]	4.71 [1.0]
3	274 [5.0]	41.1 [1.4]	8.75 [4.2]	8.70 [3.3]
4	254 [3.9]	37.5 [3.2]	6.63 [3.2]	7.92 [2.5]
5	246 [6.4]	37.1 [1.5]	4.09 [1.5]	6.39 [2.3]
6	142 [2.0]	30.7 [3.3]	5.06 [2.2]	7.90 [2.4]
7	105 [2.7]	29.4 [1.4]	2.09 [0.53]	3.65 [0.95]
8	154 [3.9]	28.8 [1.6]	2.46 [0.62]	4.42 [1.3]

Figure 2a–c shows the normalized distribution of pore and particle size for each PTL as well as the Pearson correlation coefficients of the average morphological property pairs. The property distribution functions are plotted as true fits to a log-normal power density function, with the non-normalized distribution shown in Figure S1.^26^ While the average pore and particle radii ranged from 2 to 15 μm across samples, these two properties were generally very similar to each other for each PTL. The correlation coefficients for all three property pairs are greater than 0.9, with the pore size and particle size in particular exhibiting almost perfect correlation. This indicates that porosity, pore size, and particle size are all closely correlated for these structures, though the individual properties affect performance in slightly different ways. In other words, across the PTLs studied here, fine feature size generally correlates to low porosity and coarse feature size generally correlates to high porosity. PTLs with fine features and high porosity and/or coarse features and low porosity could exist but are not readily available at this time.

**Figure 2:**
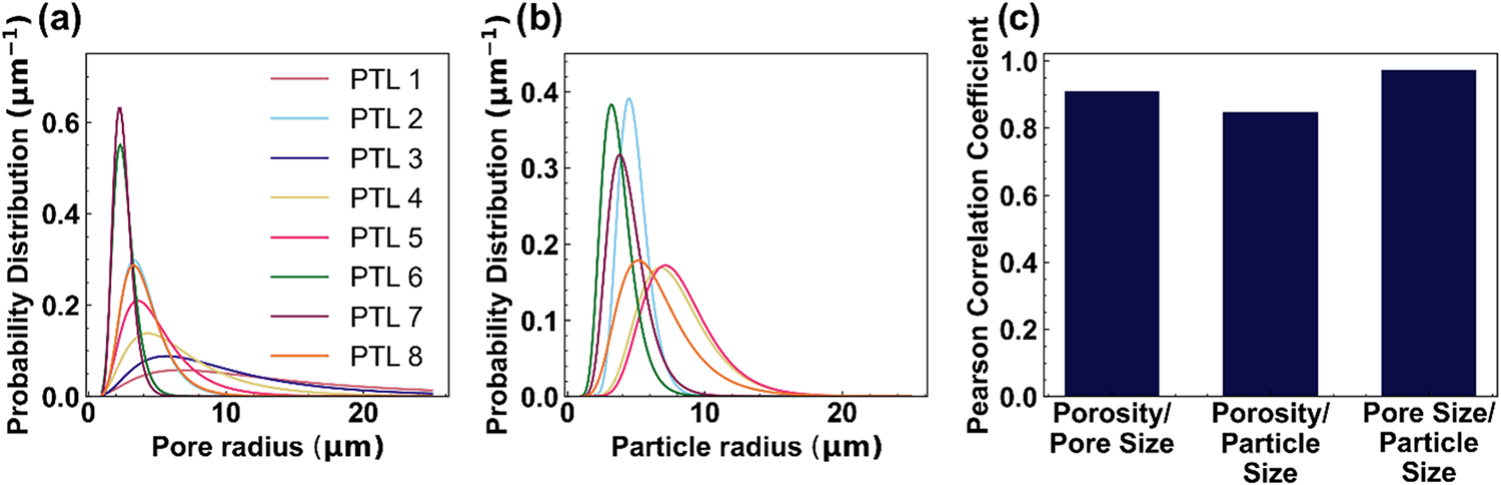
(a) Pore and (b) particle size distributions for the PTLs studied in this work (shown as log-normal power density function fits), as well as (c) Pearson correlation coefficients of the morphological property combinations (porosity, average pore size, and average particle size).

### 3.2 Beginning of Test Performance

Figure 3 shows the BOT performance of PEMWE cells using the PTLs tested in this work. Figures 3a and 3b show the cell voltages and HFR-free cell voltages of the cells with 0.4 mg_Ir_ cm^–2^ and 0.1 mg_Ir_ cm^–2^ anodes, respectively. Similarly, Figures 3c and 3d show the high frequency resistances (HFR) of those cells; these HFR values were used to obtain the HFR-free curves shown in Figures 3a and 3b. In general, Figure 3a shows that the PTL morphological differences result in minimal performance variation when using the higher anode loading. The differences in cell voltages are almost entirely driven by differences in the HFR (Figure 3c), as demonstrated by the fact that the HFR-free curves all collapse in Figure 3a. In contrast, Figure 3b shows a wide range of cell voltages when using the lower anode loadings, with a span of ∼440 mV at 4 A cm^–2^. As indicated by the HFR-free curves and HFR (Figure 3d), the performance variation is due to both HFR differences and kinetics. This is likely due to variations in the total area of electrical contact between the ACL/PTL which can affect both contact resistance (which contributes to HFR) and Ir utilization (which contributes to HFR-free voltage). The number of electrical contact points and/or the quality of these contact points is directly affected by the PTL morphologies, which is clearly important at ultralow Ir loadings.

**Figure 3:**
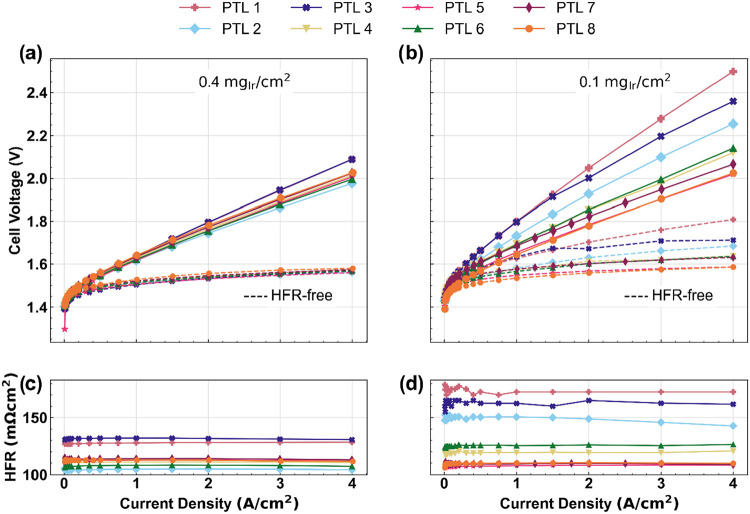
Beginning of life cell performance using the PTLs tested in this work: voltage and HFR-free voltage curves for CCMs loaded with (a) 0.4 mg_Ir_ cm^–2^ and (b) 0.1 mg_Ir_ cm^–2^; HFRs of CCMs loaded with (c) 0.4 mg_Ir_ cm^–2^ and (d) 0.1 mg_Ir_ cm^–2^.

Figure 4 shows key electrochemical performance indicators (KPIs) from the BOT diagnostics plotted against various morphological properties of the PTLs. In this work, the chosen KPIs are the average HFR (top row) and the HFR-free cell voltage at 4 A cm^–2^ (middle row). Only the 0.1 mg_Ir_ cm^–2^ data are shown here, as the data in Figure 3a indicate that the influence of the PTL properties are not as significant at the higher loading. The 0.4 mg_Ir_ cm^–2^ data are shown in Figure S2 of the Supporting Information (SI) for completeness. Note that PTL thickness was investigated but is not presented here as no significant correlations were observed. The KPIs help clarify the information contained in Figure 3, and are used to identify structure-properties-performance relationships. The horizontal error bars represent ± one standard deviation from the average PTL properties, extracted from the distributions in Figure 2. Lines of best fit are also included, where the coefficient of determination, R^2^, indicates the extent to which variation in the *y* axis variable is accounted for by the variation in the *x* axis variable. A higher R^2^ value indicates a stronger correlation between the property and KPI.

**Figure 4:**
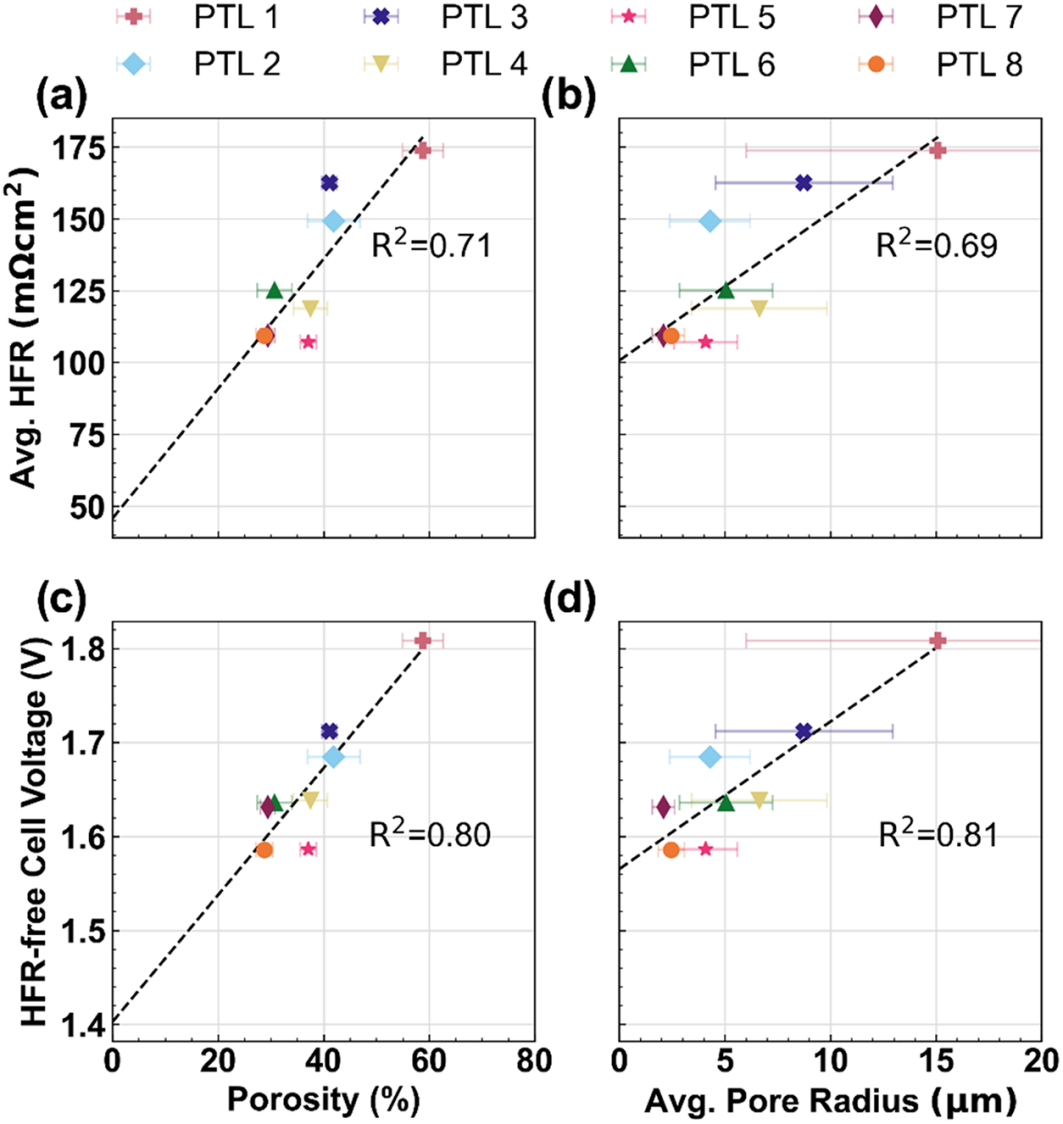
Key electrochemical performance indicators (for cells with 0.1 mg_Ir_ cm^–2^) plotted vs PTL morphological properties. The top row shows the average HFR vs (a) porosity and (b) average pore radius; the bottom row shows the HFR-free cell voltage (at 4 A cm^–2^) vs (c) porosity and (d) average pore radius.

Figures 4a and 4b, in the top row, indicate a moderate correlation between the HFR and the PTL morphology. For example, Figure 4a demonstrates that 71% (i.e., a coefficient of determination of 0.71) of the variation in HFR between samples can be attributed to the PTL porosity. This is intuitive, as the porosity (which is complementary to the solid volume fraction) represents the amount of contact area available for conducting electrons away from the anode catalyst layer. Denser PTLs also result in a more uniform distribution of ionic current in the membrane. Figure 4b shows a similar relationship (0.69) for the average pore radius, which is likely due to the strong correlation between porosity and pore size distribution. Similar, but weaker correlations are present for 0.4 mg_Ir_ cm^–2^ data (Figure S2a-b), suggesting that the PTL/catalyst layer interface still contributes to the HFR at higher loadings, but the thicker catalyst layer is more effective at distributing the current.^17^

Figures 4c and 4d show the HFR-free voltages at 4 A cm^–2^ as a function of PTL morphology. The HFR-free voltages represent the contributions of anode kinetics, catalyst utilization, and residual overpotentials to the total cell voltage. Figures 4c and 4d indicate strong correlations between the HFR-free voltage and the PTL porosity/pore radius, with coefficients of determination of 0.80 and 0.81, respectively. The average PTL pore radius more significantly impacts the HFR-free voltage than it does the HFR, indicating that PTL morphology does alter the electrochemical performance of the anode (and not just ohmic losses, as might be expected from a component that is not catalytically active). Since the ACLs are the same for each cell, we attribute the improved HFR-free voltages to increased Ir utilization stemming from the increased interfacial contact area from PTLs with lower porosities and smaller pore radii. This is consistent with prior related studies^18,19,21^ which also note that the anode performance is affected by the ACL/PTL interface, rather than the properties of the ACL alone.

Figure S3 shows the electrochemical KPIs as a function of average PTL particle radius for cells with 0.4 and 0.1 mg_Ir_ cm^–2^. Weaker correlations are observed for the PTL particle size, indicating that this parameter, individually, does not affect the HFR and HFR-free cell voltage as much. In summary, the data in Figure 4 show that the average pore radius most strongly impacts the anode performance, likely because it represents the distance electrons must travel in-plane in the ACL before they can be conducted into the PTL. However, it is useful to note that the porosity can also be used as a representative indicator, since porosity is typically easier to measure compared to the pore radius.

Tafel slopes, plotted in S4, were also investigated as a measure of the impact of the PTL on kinetics. While moderate correlations with the morphological properties exist, with R^2^ values ranging from 0.39 to 0.54 for 0.1 mg_Ir_ cm^–2^ samples, the trends are weaker compared to those discussed and shown in Figure 4. Again, the strongest correlation is observed for the average pore radius, with Tafel slopes increasing with increasing PTL porosity, pore radius, and particle radius. While the Tafel slope is typically used to identify and describe electrochemical reaction mechanisms, it can also be an indicator of catalyst utilization. The true Tafel slope (e.g., as results from a simplification of the Butler–Volmer equation) represents kinetic overpotentials when the electrochemical current distribution is uniform throughout the catalyst layer.^22,27^ While we do not expect any differences in the kinetic mechanism of the OER in this case, the apparent Tafel slope, as extracted from the HFR-free cell voltage and plotted in Figure S4, can be affected by in-plane ohmic losses and will appear higher when the catalyst layer resistance is higher and the Ir utilization is lower. However, at the low current densities (<100 mA cm^–2^) used for Tafel analysis, the variations in utilization are expected to be small, resulting in the weaker correlations in Figure S4. In contrast, variations in utilization grow at larger current densities,^18^ creating the stronger correlations in the HFR-free cell voltage at 4 A cm^–2^ shown in Figure 4. Figure S5, which shows the Tafel plots, demonstrates this effect, as the Tafel fits converge at low currents for both 0.4 and 0.1 mg_Ir_ cm^–2^ cells. This indicates that the Ir utilization across samples is similar at low current densities but diverges significantly at higher current densities due to varying catalyst layer resistance losses.

The results in Figures 3 and 4 combined indicate that PTLs with lower porosities, smaller pores, and smaller particle radii can enable good PEMWE cell performance with low-loaded anodes. The PTLs that exhibited the lowest cell voltages at 0.1 mg_Ir_ cm^–2^, PTLs 5, 7, and 8, featured the lowest porosity and the smallest pore radii. For the best performer, PTL 8, the cell voltage at 4 A cm^–2^ was 2.020 V regardless of whether the anode loading was 0.1 mg_Ir_ cm^–2^ or 0.4 mg_Ir_ cm^–2^.

In contrast to Figure 4, Figure S2 shows that the KPIs are only weakly correlated to PTL morphology at higher anode loadings (0.4 mg_Ir_ cm^–2^). This indicates that the cell performance at higher loadings is relatively independent of PTL properties. Figure S6, which shows the ACL sheet resistance vs Ir loading, provides additional context to this observation. At loadings of ∼0.4 mg_Ir_ cm^–2^ and above, the in-plane catalyst layer sheet resistance is low and only weakly dependent on loading. At lower Ir loadings, the catalyst layer resistance increases exponentially. For example, the electronic resistance at 0.1 mg_Ir_ cm^–2^ is more than ten times higher than at 0.4 mg_Ir_ cm^–2^, when it may be expected to only differ by a factor of 4 assuming coating thickness is proportional to loading. Figure S6 indicates that catalyst layers with dry sheet resistances less than ∼10 kΩ sq^–1^ (or ≥5 S m^–1^) will function well regardless of PTL, whereas more resistive catalyst layers will require denser PTLs to facilitate electron conduction. A similar threshold, ∼10 to 100 S m^–1^ was determined in a recent modeling study,^17^ but note that these values are not directly comparable as the results in Figure S6 were obtained from a dry, uncompressed catalyst layer.

These results suggest that an additional phenomenon beyond loading reduction causes the strongly increasing sheet resistance. At ultralow loadings, the unsupported IrO_*x*_ network is likely discontinuous and may form “islands” of poorly connected material.^17^ Note that sheet resistance depends on the ink dispersion, I/C ratio, and the catalyst material itself, and is subject to change for other catalyst layer recipes/architectures. Nonetheless, PTLs with high porosities and/or large pores force electrons to travel longer distances in the in-plane direction within the ACL, which increases ohmic losses and lowers catalyst utilization. Both effects manifest as higher HFR-free voltages, as demonstrated in previous work.^18,21,22^ In contrast, the 0.4 mg_Ir_ cm^–2^ loading is above the threshold for good conductivity, indicating that the Ir network is mostly continuous and can sustain in-plane electron transport at lower ohmic losses.

Notably, the results in Figures 3 and 4 suggest that the best possible PTL would feature a porosity approaching 0%, i.e., a solid sheet. Of course this cannot be true, as a fully dense PTL would block liquid water from reaching the ACL and O_2_ gas from leaving. In such a scenario, significant mass transport losses would be observed. However, none of the performance data in Figure 3 exhibit mass transport losses at the tested current densities, and no data in the open literature have yet shown such effects at reasonable water flow rates and when properly correcting for anode utilization losses.^22,28^ Therefore, the minimum optimal PTL porosity may be even less than 29%, the lowest porosity studied in this work. Future efforts will focus on finding this minimum and investigating the trade-off between improved electrical contact area and the onset of mass transport losses.

### 3.3 Durability Performance

A subset of the PTLs (1, 3, 4, and 6) were selected for durability testing at anode loadings of both 0.4 mg_Ir_ cm^–2^ and 0.1 mg_Ir_ cm^–2^. These PTLs feature porosities that nearly span the full range studied in this work – the data obtained from these tests are shown in Figure 5 and Figure 6, respectively. Durability testing consisted of a 1000-h 2 A cm^–2^ galvanostatic hold, an industrially relevant current density, with intermittent collection of polarization curves and EIS (using a potentiostat/galvanostat). Figures 5 and 6 show both continuous voltage vs time traces as well as voltage breakdown metrics (cell voltage, HFR, and HFR-free cell voltage) from the intermittent diagnostics. A direct comparison between the two loadings at BOT and end of test (EOT) can be seen in Figure S7. In Figures 5a and 6a, the cell voltages at 2 A cm^–2^ obtained from intermittent polarization curves are marked using stars of the corresponding color for each cell. These data points help illustrate the extent of reversible losses^24^ by comparing the cell voltage before and after interruption of the 2 A cm^–2^ hold. Such interruptions are the result of switching from test station power supply to potentiostat/galvanostat control and the measurement of the polarization curve. Cell voltages at 2 A cm^–2^ from the diagnostic polarization curves are consistently lower than the cell voltages during the long duration hold. This phenomenon is often observed in PEMWE durability testing^24^ and may be related to IrO_*x*_ redox transitions when the cells are depolarized and repolarized or other effects on the cell state.

**Figure 5:**
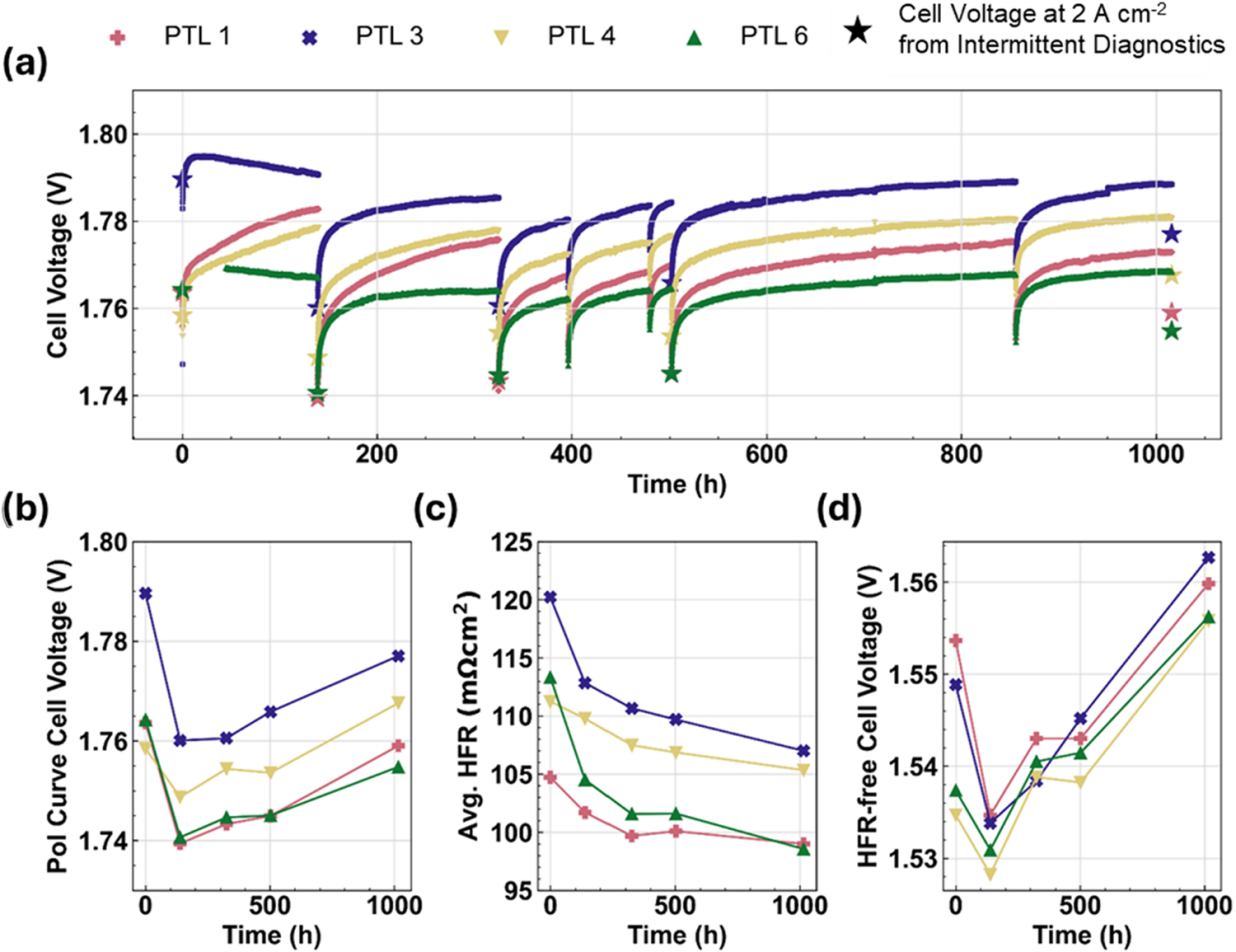
Durability testing results for 0.4 mg_Ir_ cm^–2^ cells: (a) cell voltage vs time during a 2 A cm^–2^ galvanostatic hold, (b) cell voltage at 2 A cm^–2^ from intermittent diagnostic polarization curves, (c) average HFR from intermittent polarization curves, and (d) HFR-free cell voltage at 2 A cm^–2^ from intermittent polarization curves.

**Figure 6:**
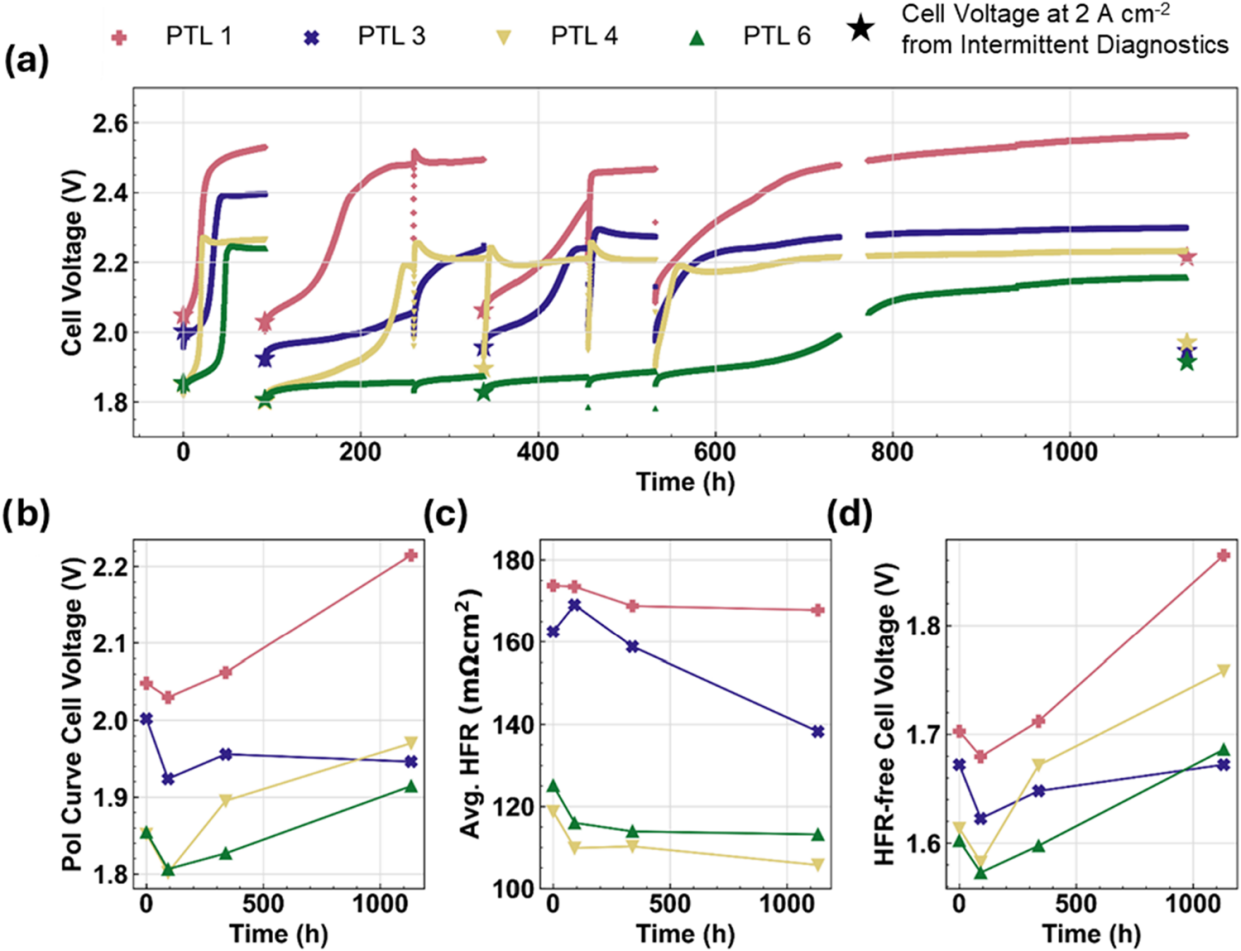
Durability testing results for 0.1 mg_Ir_ cm^–2^ cells: (a) cell voltage vs time during a 2 A cm^–2^ galvanostatic hold, b) cell voltage at 2 A cm^–2^ from intermittent diagnostic polarization curves, (c) average HFR from intermittent polarization curves, and (d) HFR-free cell voltage at 2 A cm^–2^ from intermittent polarization curves.

Figure 5a shows the evolution of cell voltages (at 2 A cm^–2^) over time for cells with anode loadings of 0.4 mg_Ir_ cm^–2^. The cell voltages start between 1.76 and 1.79 V and remain within this range for the entire duration of the test. PTL 6 (green) exhibits the lowest voltage over the entire duration, though all cell voltages remain within 20 mV of each other, regardless of the PTL used. Figure 5b shows that the cell voltages at 2 A cm^–2^ from the diagnostic polarization curves decrease during the first ∼100 h of testing, reflecting an extended break-in period, followed by a steady increase for the remainder of the 1000-h test. Figures 5c and 5d show that both the average HFR and the 2 A cm^–2^ HFR-free cell voltages decrease during the initial period as well. However, while the HFRs decrease monotonically over the entire 1000-h test, the HFR-free cell voltages monotonically increase after the first ∼100 h.

Figure 6 shows similar durability testing data for cells with anode loadings of 0.1 mg_Ir_ cm^–2^. The cell voltages in Figure 6a start between 1.8–2.0 V; 90–200 mV higher than their 0.4 mg_Ir_ cm^–2^ counterparts in Figure 5a. Most notably, an “S-shaped” transition or runaway voltage occurs in the first 50 h in which the cell voltages rapidly increase before plateauing at higher values. The total voltage changes of the transitions range from 390 mV for PTL 6 to 480 mV for PTL 1. Despite this large change, the cell voltages at 2 A cm^–2^ from the diagnostic polarization curves collected at 90 h are lower than the BOT voltages. In other words, the S-shaped transition is largely reversible.

Following the 90-h diagnostics, the cell voltages for PTLs 1, 3, and 4 again undergo a similar transient behavior with two key differences: (i) the onset of the transition takes longer (>100 h compared to 24–48 h), and (ii) the changes in voltage are smaller (∼335 to 450 mV this time). Furthermore, PTL 6 does not immediately undergo this transition and instead exhibits a continuous, slow voltage increase (i.e., “normal” durability behavior) for more than 600 h before eventually undergoing the S-shaped transition again. The voltage change of the second transition for PTL 6 is about 260 mV, significantly less than the 390 mV observed the first time. These significant voltage increases may correlate to total Ir dissolution, with the OER catalyzed by the Pt coating on the PTLs. However, this hypothesis would not explain the reversibility in Figure 6.

Figures 6c and 6d show the average HFR and HFR-free cell voltage (at 2 A cm^–2^) over time for the 0.1 mg_Ir_ cm^–2^ samples, which exhibit similar trends to those in Figures 5c and 5d. The HFRs mostly decrease throughout the 1000-h test. The HFR-free voltages exhibit a decrease during the first ∼100 h, followed by a monotonic increase for the remainder of the test. The data from the intermittent diagnostics appear largely analogous to those in Figure 5 despite the drastically different behavior during the current holds. These details indicate that the S-shaped transitions are largely reversible phenomena.

Figure 7 identifies two trends in the voltage decay rates for the 0.4 mg_Ir_ cm^–2^ samples. Figure 7a shows the voltage decay rate vs PTL porosity, and Figure 7b shows the voltage decay rate vs the HFR-free cell voltage (at 2 A cm^–2^) for the 0.1 mg_Ir_ cm^–2^ samples using the same PTLs. The relationships with average pore and particle sizes as well as the analogous 0.1 mg_Ir_ cm^–2^ data are shown in Figures S8 and S9, respectively. Voltage decay rates were calculated by linearly fitting the cell voltages at 2 A cm^–2^ from the intermittent polarization curve diagnostics; this approach was established in a prior work^24^ to specifically address the irreversible losses occurring in the cell. BOT data points were excluded when determining decay rates due to the decrease in cell voltages during the first ∼100 h, as seen in Figures 5b and 6b.

**Figure 7:**
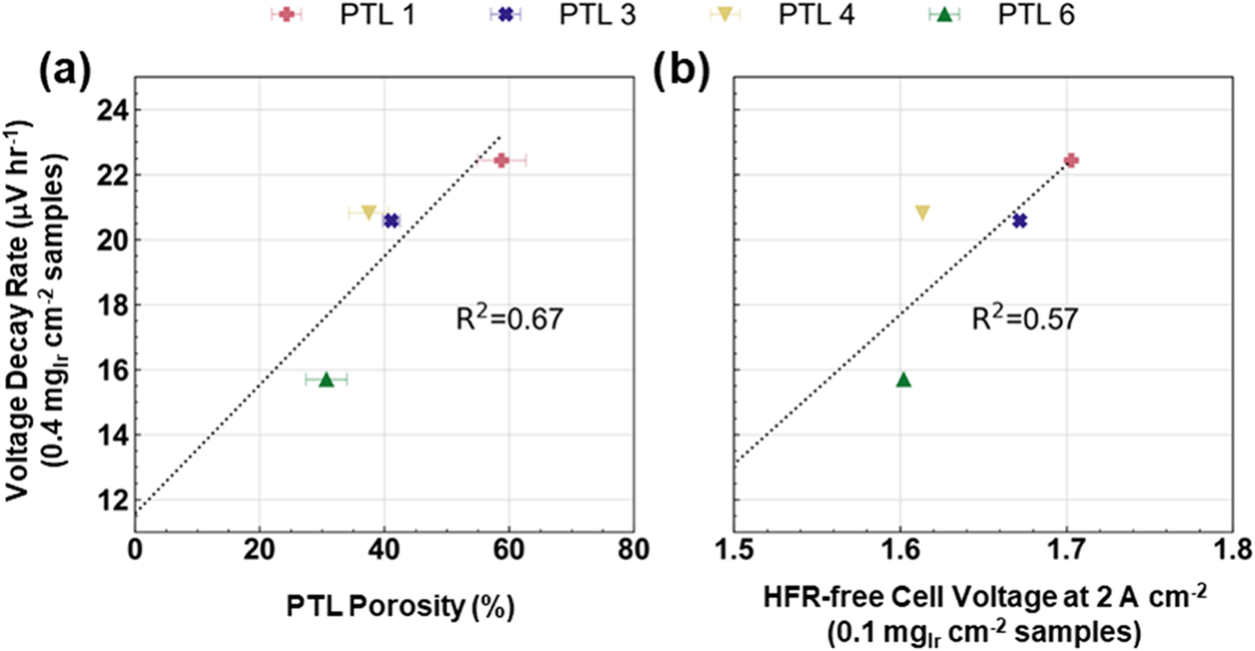
Voltage decay rates of 0.4 mg_Ir_ cm^–2^ samples during durability testing vs (a) PTL porosity and (b) HFR-free cell voltages (at 2 A cm^–2^) of 0.1 mg_Ir_ cm^–2^ samples using the same PTLs.

Figure 7a shows that the cell durability is significantly impacted by PTL porosity even at high loadings, with the lowest porosity PTL exhibiting significantly lower degradation rates: ∼16 μV h^–1^ for PTL 6 compared to 22 μV h^–1^ for PTL 1. This is complementary to the BOT performance data shown in Figure 4. Notably, the PTL with the lowest degradation rate, PTL 6, was also the most resistant to the S-shaped transitions during the 1000-h hold in Figure 6a and exhibited some of the best BOT performance in Figures 3 and 4. This indicates a link between BOT performance and durability at both high and low loadings that is strongly affected by the PTL morphology. Figure 7b shows that improved durability at higher loadings (0.4 mg_Ir_ cm^–2^) is also linked to better BOT performance at 0.1 mg_Ir_ cm^–2^. We propose that this is because the 0.1 mg_Ir_ cm^–2^ samples magnify Ir utilization issues that are latent in the 0.4 mg_Ir_ cm^–2^ samples, obscured by the mitigating effects of the higher loading. During the electrode ink deposition process, as the Ir loading is increased the general morphology of the Ir catalyst particles/aggregates stays the same, but the addition of more catalyst increases the likelihood of interparticle connections. This minimizes utilization losses in BOT cell performance. During long-term testing, as the Ir dissolves and interparticle connections disappear, utilization issues can resurface which explains the link between 0.1 mg_Ir_/cm^2^ BOT performance and 0.4 mg_Ir_/cm^2^ durability performance. The results in Figures 3–7 show that by faciliting electron transport, denser PTLs increase Ir utilization which not only improves the BOT performance of CCMs with ultralow Ir loadings, but also improves the durability of CCMs with higher loadings.

### 3.4 Post Test Microscopy

After testing, the ACL regions of CCM cross sections from the samples in Figures 6 were analyzed using STEM-EDS. These cross sections are shown in Figure 8: the left column (Figures 8a and 8c) shows a pristine 0.1 mg_Ir_ cm^–2^ ACL at two different magnifications, and right column shows ACL cross sections for PTL 1 after the 1000-h test. Analogous images for PTLs 3, 4, and 6 are included in Figure S10. The post-test samples all feature bright white lines corresponding to the Pt coatings of the PTLs which delaminated after cell disassembly. The ACL regions of interest are located underneath the Pt. Figures 8a and 8c show that the pristine ACLs feature amorphous, roughly spherical Ir aggregates about 50–100 nm in diameter only in the ACL. The post-test cross section in Figure 8b indicates the presence of Ir “bands” in membrane near the ACL interface. These bands consist of Ir that experienced dissolution and subsequent redeposition/immobilization in the membrane. Some of the Ir band may be electrochemically active (if it is electrically connected), but given the poor performance in Figure 6 the majority is likely inactive.

**Figure 8:**
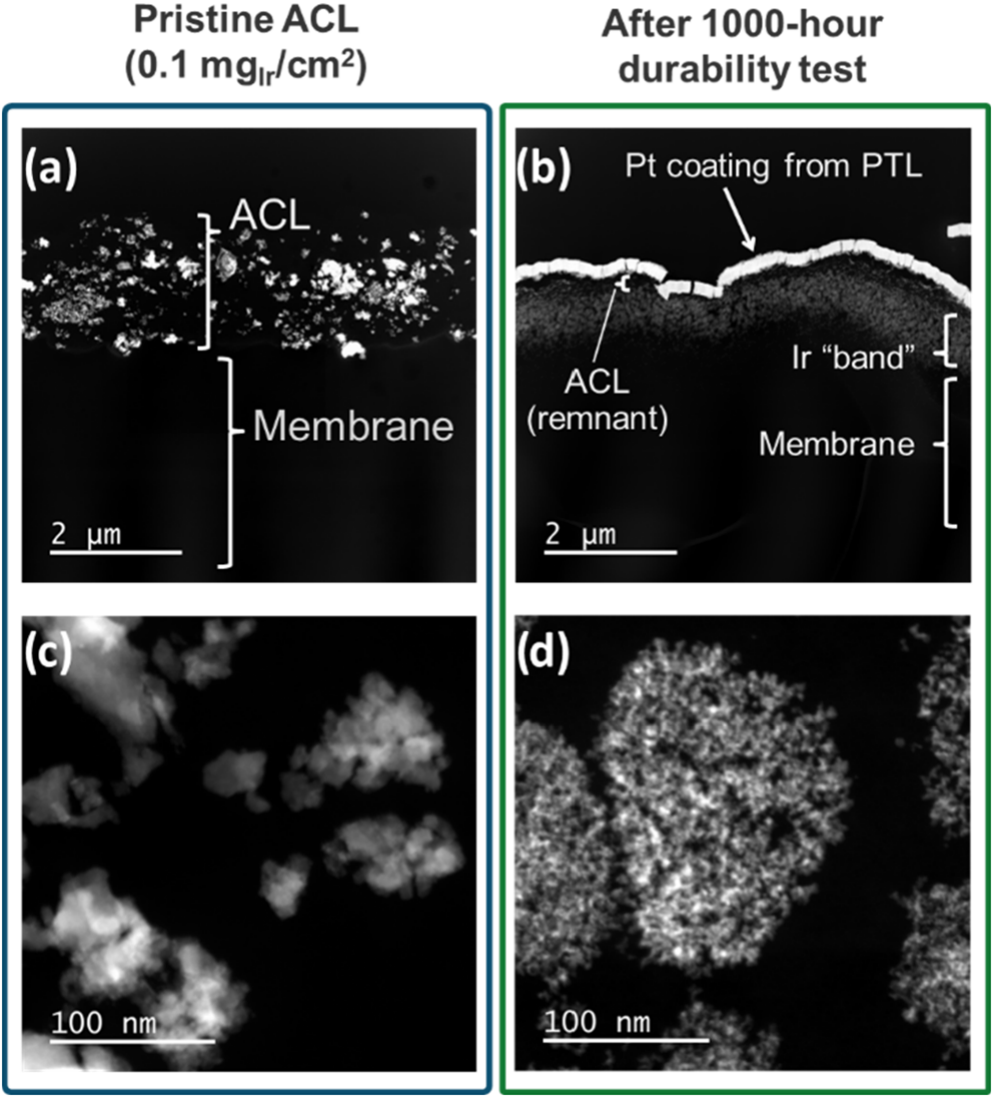
High-angle annular dark-field scanning transmission electron microscopy (HAADF-STEM) images of the 0.1 mg_Ir_ cm^–2^ anode catalyst layers before (a and c) and after (b and d) 1000 h of testing (PTL 1 shown here, PTLs 3, 4, and 6 are shown in the SI).

Compared to Figure 8c, Figure 8d shows that the IrO_*x*_ networks significantly changed due to long-term testing. The IrO_*x*_ appears to form nanocrystalline phases with much smaller particles after long-term operation. The morphology of the IrO_*x*_ also appears to have changed from discrete spherical aggregates to a more dispersed structure. Figure S10 shows that no clear differences are apparent between the different PTLs. Even PTL 6, which appeared to resist the runaway voltages seen in Figure 6a, displays similar nanoscale IrO_*x*_ morphology to the other PTLs. These images demonstrate that despite exhibiting normal performance during the (EOT) diagnostics, the catalyst layer structures were significantly impacted by the 1000-h testing.

Figure 9 shows STEM-EDS images of a cathode catalyst layer (CCL) cross section from the PTL 1 sample shown in Figures 6 and 8. Similar images for PTLs 3, 4, and 6 are included in Figure S11. The elemental mapping from STEM-EDS reveals strong signals from Ti, Pt and Ir in the cathode. While Pt is present as the intended cathode catalyst material, the Ti and Ir originate from the anode and ostensibly dissolved and migrated across the membrane to reach the CCL. These Ir and Ti deposits have been observed and analyzed in more detail in a previous investigation,^5^ where it was found that the Ir deposits on the Pt catalyst particles (but is unlikely to impact hydrogen evolution kinetics) while the Ti deposits as an oxide in isolated particles, that are unlikely to directly impact the catalyst function. Figure S11, like Figure S10, shows no significant differences across the different PTL materials. While the Ir and Pt appear uniformly distributed throughout the CCL, the Ti is more concentrated on the GDL-facing side of the CCL. Since the Ti and Ir traversed the membrane as cations (e.g., Ti^2+/3+^ and Ir^3+^), they must have infiltrated the CCL through the ionomer network. This is problematic, since this means that they displaced an amount of H^+^ equivalent to their charge prior to deposition in solid forms. This phenomenon can have negative performance impacts, as will be discussed in the next section.

**Figure 9:**
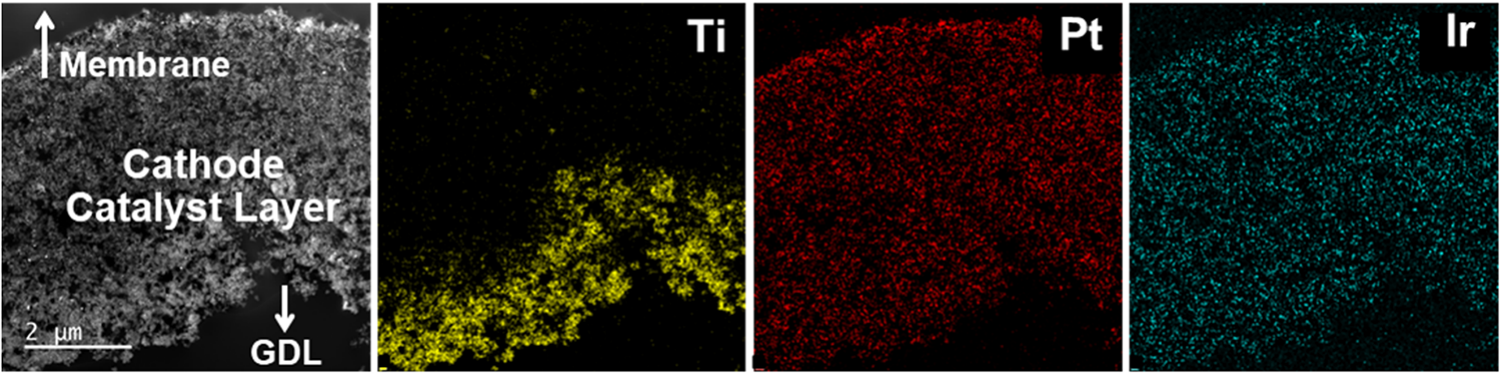
Scanning transmission electron microscopy energy dispersive X-ray spectroscopy (STEM-EDS) images of the cathode catalyst layers (0.1 mg_Ir_ cm^–2^ anodes) after 1000 h of testing (PTL 1 shown here, PTLs 3, 4, and 6 are shown in the SI).

### 3.5 Runaway Voltage

The 0.1 mg_Ir_ cm^–2^ CCMs exhibited unexpected behavior during durability testing, featuring a characteristic S-shaped runaway cell voltage vs time profile. It is worth investigating this effect as increased understanding may enable the operation of ultralow Ir loaded CCMs. Replicate 2 A cm^–2^ hold tests were performed on additional 0.1 mg_Ir_ cm^–2^ CCMs to gauge the reproducibility of the effect. One additional sample was tested with PTL 1 and two with PTL 6. Figures S12 and S13 show the cell voltage vs time for these cells, which demonstrated similar performance compared to Figure 6.

Table 2 shows quantitative analysis of the migrated Ir within the replicate CCMs, both in the Ir band and in the cathode. Two samples (PTL 1 Repeat #1, PTL 6 Repeat #1) exhibited runaway voltages and both show approximately the same percentage of total Ir in the Ir band (∼70%), despite different test durations (190 h vs 457 h). The third sample (PTL 6, Repeat #2) did not exhibit a runaway voltage. This sample featured much less Ir in the Ir band (42%) than the other two samples, which had both a longer (PTL6, Repeat #1) and a shorter (PTL1, Repeat #1) experimental duration. These results suggest that the runaway voltage phenomenon is correlated to the amount of Ir dissolving from the bulk of the catalyst layer. However, while dissolution does lead to more Ir in the Ir band, it alone does not explain the reversibility observed in Figure 6.

**Table 2:** Sample Information and Quantification of Ir Migration for Replicate Durability Tests Using 0.1 mg_Ir_ cm^–2^ CCMs

			% of Total Ir:		
sample ID	voltage runaway	test duration (hours)	in anode	in Ir band	in cathode
PTL 1 (Repeat #1)	Yes	190	25.6	69.6	4.8
PTL 6 (Repeat #1)	Yes	457	21.7	70.5	7.8
PTL 6 (Repeat #2)	No	261	52.8	42.4	4.8

Table 2 also shows the amount of Ir in the cathode. In contrast to the Ir band, the amount of Ir in the cathode appears to be more closely tied to the test duration than the presence of voltage runaway. For example, PTL 1 Repeat #1 and PTL 6 Repeat #2, which were on test for a similar duration, both featured 4.8% of total Ir in the cathode even though PTL 1 Repeat #1 experienced the runaway voltage and PTL 6 Repeat #2 did not.

Figure 10 shows results from a PEMWE reference cell experiment. Observing the changes in the anode and cathode voltages over time provides useful insight into the processes occurring at each electrode. The cell was operated at 3 A cm^–2^ for 10 h using PTL 1 and an anode loading of 0.1 mg_Ir_ cm^–2^. Polarization curves with simultaneous EIS were also collected before and after this short hold. Figure 10a shows the total cell voltage over time in the reference electrode experiment, which increased quickly from 1.96 V at BOT to plateau around 2.5 V. The S-shaped transition in the cell voltage was consistent with the results of Figure 6 as well as Figures S12 and S13. Notably, Figures 10b and 10c indicate that both the anode and cathode potentials contribute relatively equally to the increase in total cell voltage. Therefore, the runaway voltage appears to arise from processes occurring in both the anode and cathode catalyst layers. For example, Ir and Ti dissolution from the anode and subsequent transport to the cathode is one possible mechanism creating the runaway cell voltage. As shown in Figure 8 and Table 2, the CCMs with ultralow anode loadings experience significant Ir dissolution from the thin catalyst layer, which would result in an increase in the anode catalyst layer overpotential as catalyst active sites are lost.^5,29–31^ Additionally, studies investigating cation contamination in PEMWE cells have shown similar S-shaped voltage profiles.^32–34^ In cation contamination experiments, this profile was attributed to a transition from the acidic to the alkaline HER mechanism due to pH increases in the cathode as protons are replaced by inert cations. Thus, the observations in Figures 8–10, supported by discussion in the literature, suggest that Ir and Ti dissolution from the anode components and deposition at the cathode could cause the runaway cell voltage phenomenon observed here.

**Figure 10:**
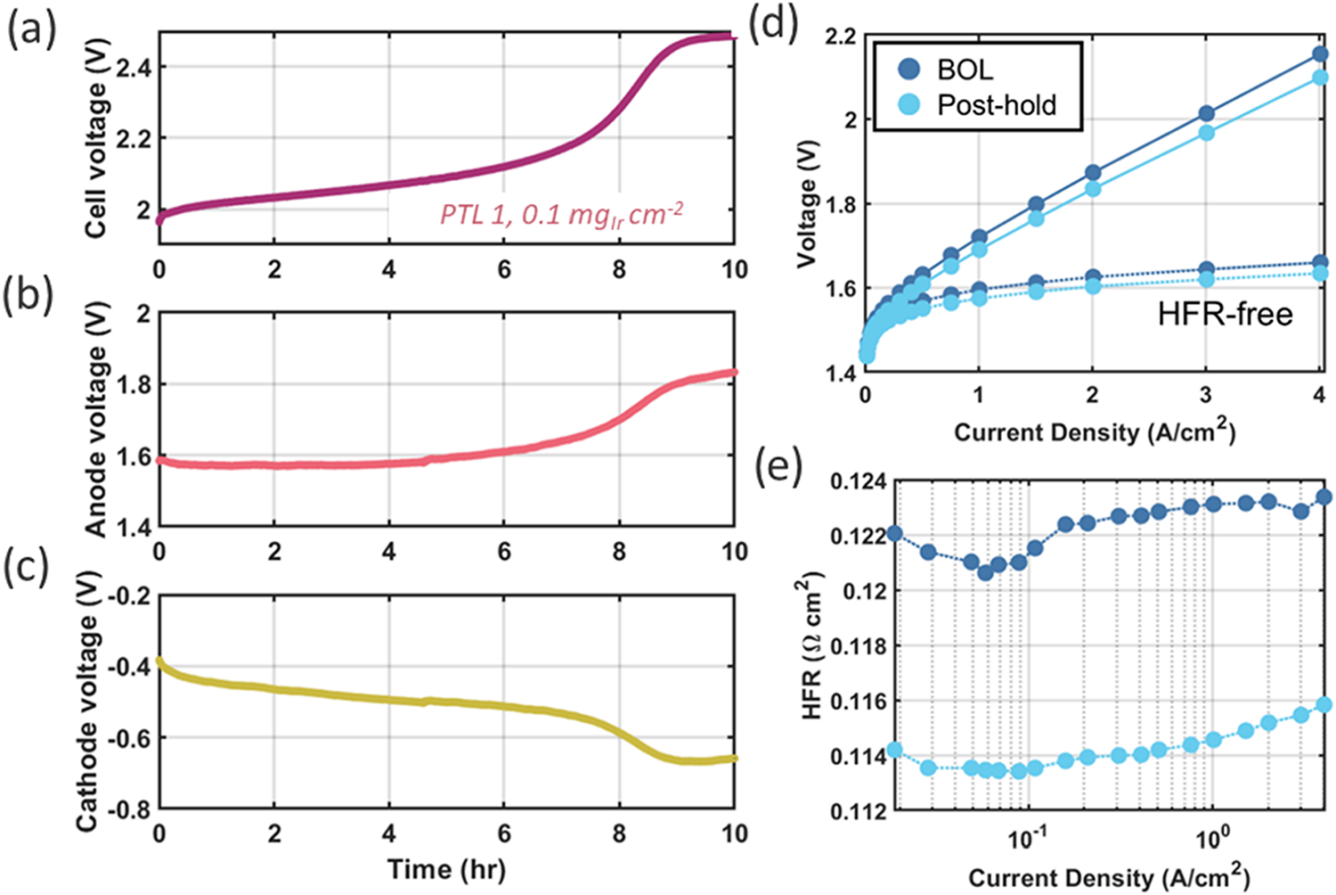
Reference electrode measurements for a 0.1 mg_Ir_ cm^–2^ cell using PTL 1 depicting (a) total cell voltage measured during a 3 A cm^–2^ current hold, (b) isolated anode voltage, and (c) isolated cathode voltage. Also shown are (d) polarization curves with total and HFR-free voltages and (e) corresponding HFR values from beginning of test and end of test.

Based on our observations in Figure 6, the mechanism for the voltage increase is at least partially reversible. Increases in cell voltage associated with accumulation of cationic impurities in the cathode catalyst layer have been shown to be recoverable by applying high current densities and altering cell temperature.^32^ This expectation may be consistent with some portion of the reversibility observed in Figure 6, where cell performance is initially recovered following polarization curve/impedance diagnostic measurements. However, the extent of reversibility observed in Figure 6 also suggests that some portion of these increases in anode voltage may be attributable to redox processes that can be reversed upon returning to low current densities and/or open circuit conditions during polarization curve/impedance diagnostics. Direct operando evidence of changes in anode oxidation state are difficult to obtain,^24,35^ so further experimentation or modeling is needed to understand the observed reversible runaway voltage phenomenon.

## 4 Conclusions

Tuning the PTL morphology represents an impactful and tractable strategy for improving Ir utilization, enabling good PEMWE performance and durability. In this work, we surveyed eight different commercially available PTLs and assessed their impact on the performance and durability of PEMWE cells with varying Ir loadings in the anode. We found strong correlations between the PTL porosity and average pore radius on both HFR and HFR-free voltage. PTLs with smaller pores and lower porosity lead to improved cell performance by increasing the electrical contact area at the anode catalyst layer/PTL interface and reducing catalyst layer in-plane ohmic losses. The low porosity PTLs enable high performance in cells with low anode catalyst layer conductivities, particularly when using ultralow Ir loadings (0.1 mg_Ir_ cm^–2^), which typically suffer from a poorly connected catalyst network.

In addition, cells employing denser PTLs with smaller pores/particles featured improved voltage decay rates during long-term testing, even for the higher loading cells. This was also ascribed to better electrical contact with the PTL, leading to higher Ir utilization and hence lower local anode overpotentials. Our data demonstrate a link between the BOT performance of 0.1 mg_Ir_ cm^–2^ cells to the 1000-h degradation rates of 0.4 mg_Ir_ cm^–2^ cells. This implies that short-term testing at ultralow loadings can be used as an indicator of long-term degradation at higher loadings. While future data is required to reinforce this hypothesis, such a correlation may be used to greatly accelerate the assessment of novel catalyst material durability. In other words, this correlation can be used to rapidly down-select materials for long-term testing and focus experimental resources on the most promising materials.

PEMWE cells with ultralow Ir loadings, i.e., 0.1 mg_Ir_ cm^–2^, exhibited a runaway voltage phenomenon during long-term testing. We propose that this behavior is caused by a combination of significant Ir and Ti dissolution from the anode, followed by migration to the cathode. Although all tested PTLs exhibited this effect, very low porosity PTLs appear to delay the runaway voltage. In the future, even denser PTLs than those currently commercially available could further improve durability with ultralow Ir loadings.

Note that no mass transport limitations were observed for any PTL across the tested current density range, which implies that the optimal PTL porosity may be at or lower than 29% (the lowest porosity studied in this work). However, future efforts will continue to carefully examine the potential trade-offs between improved electrical contact and mass transport limitations.

## Supplementary Material



## Notes

Additional KPI plots, polarization curves, durability data, and sheet resistance measurements (PDF)
