# Supplementary material for: Impact of Porous Transport Layer Morphology on the Performance of Proton Exchange Membrane Water Electrolyzers with Ultra-Low Iridium Loadings
Source: ACS Appl Mater Interfaces. 2026 Apr 1;18(14):20547–58. doi: 10.1021/acsami.6c01544 (PMC13088025; doi:10.1021/acsami.6c01544)
Supplement: Supplementary file 1 [file am6c01544_si_001.pdf]

Supporting Information for:

The Impact of Porous Transport Layer Morphology on the Performance of Proton Exchange Membrane Water Electrolyzers with Ultra-Low Iridium Loadings

Jacob A. Wrubel[1,*], Makenzie Parimuha[1], Sarah Blair[1], Haoran Yu[2], Jack Todd Lang[3], Abigail J. Schmeiser[1], James L. Young[1], Elliot Padgett[1], Iryna V. Zenyuk[3], and Guido Bender[1]

[1]National Laboratory of the Rockies, Golden, CO, USA
[2]Oak Ridge National Laboratory, Oak Ridge, TN, USA
[3]University of California, Irvine, Irvine, CA, USA

*Corresponding Author Email: jacob.wrubel@nlr.gov

Figure S1:

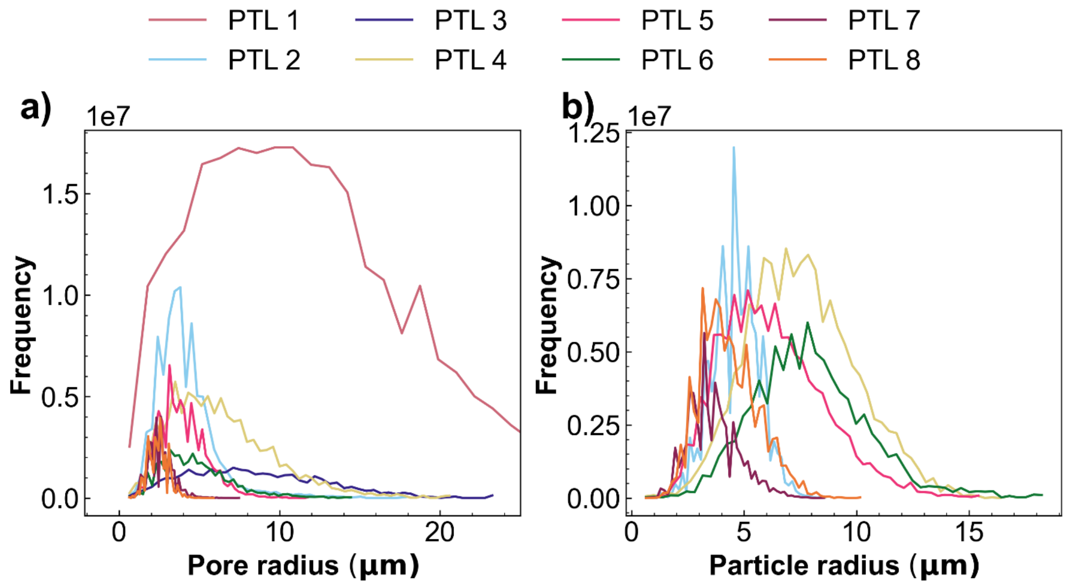

Figure S1 – Non-normalized a) pore and b) particle size distributions for the PTLs studied in this work.

Figure S2:

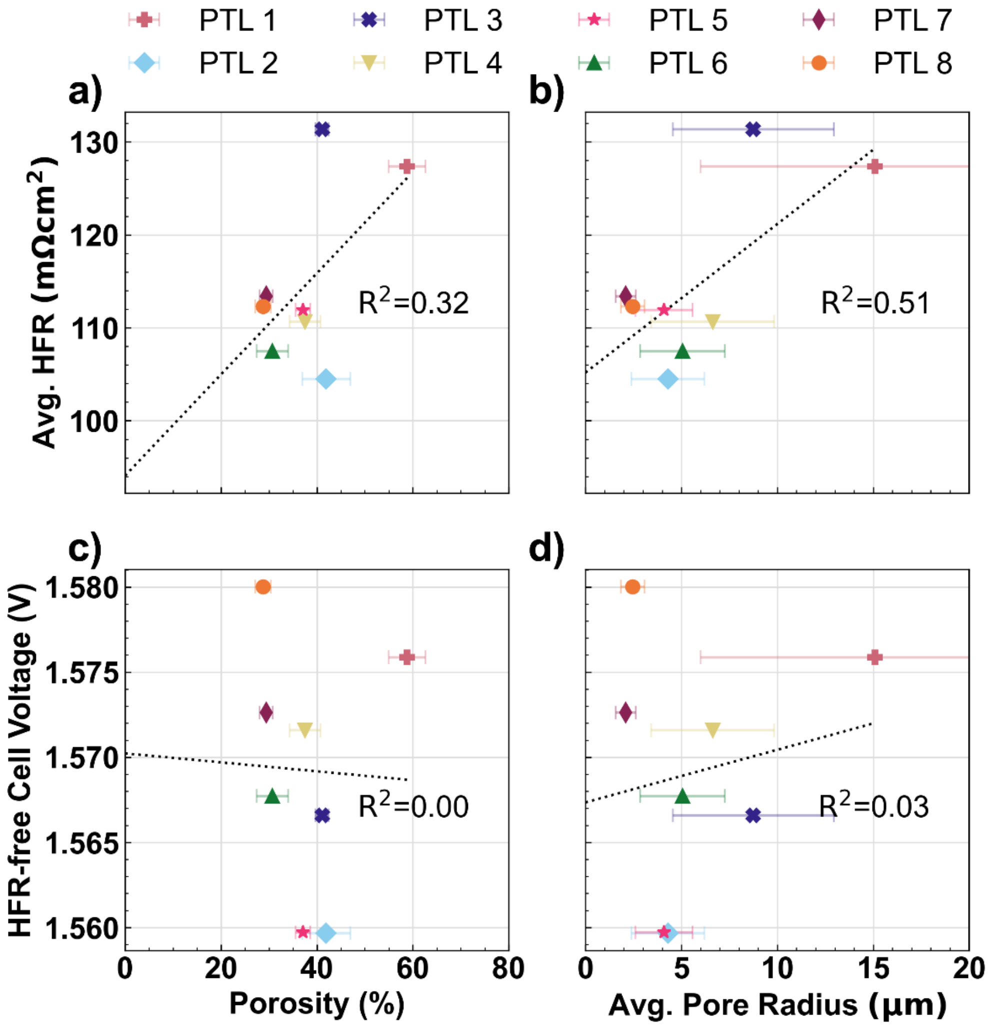

Figure S2 – Key electrochemical performance indicators (for cells with 0.4 mg_{Ir} cm^{-2}) plotted vs. PTL morphological properties. The top row shows the average HFR vs. a) porosity and b) average pore radius; the bottom row shows the IR-free cell voltage (at 4 A cm^{-2}) vs. c) porosity and d) average pore radius.

Figure S3:

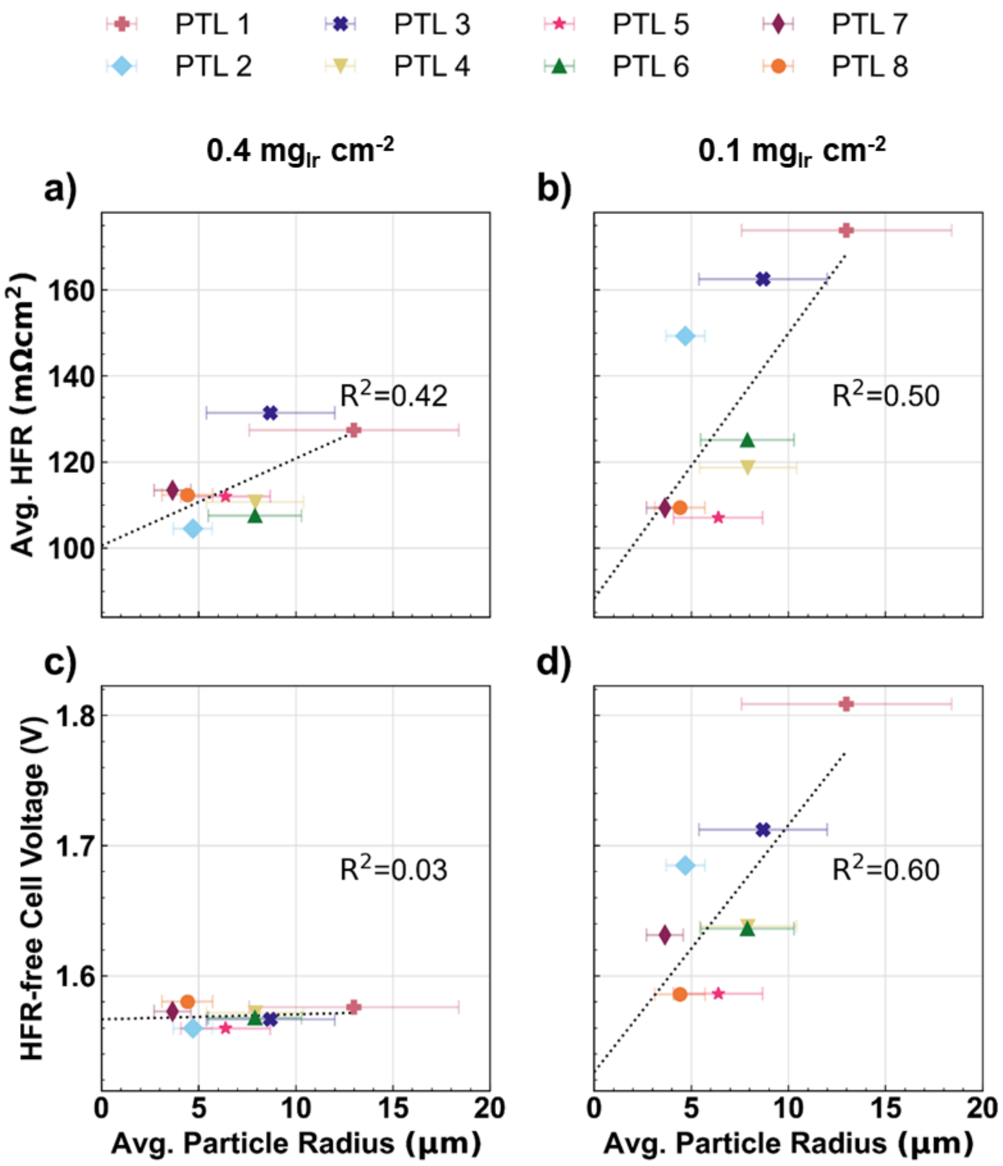

Figure S3 – Key electrochemical performance indicators plotted vs. average PTL particle size; the left column, a) and c), denotes cells with 0.4 mg_{Ir} cm^{-2} and the right column, b) and d), denotes cells with 0.1 mg_{Ir} cm^{-2}

Figure S4:

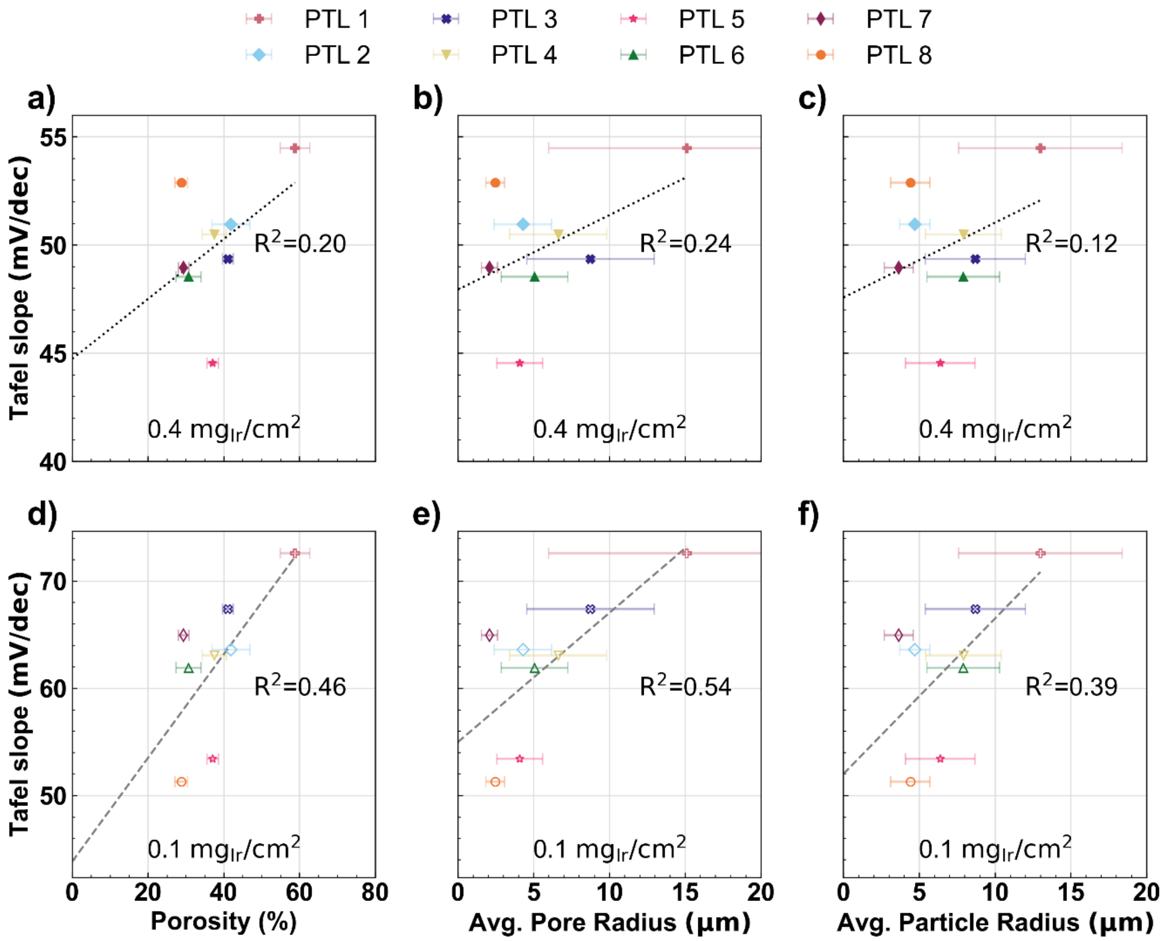

Figure S4 – BOL Tafel fits of HFR-free cell voltage vs. PTL morphological properties for a) - c) 0.4 mg_{Ir} cm^{-2} cells and d) - f) 0.1 mg_{Ir} cm^{-2} cells.

Figure S5:

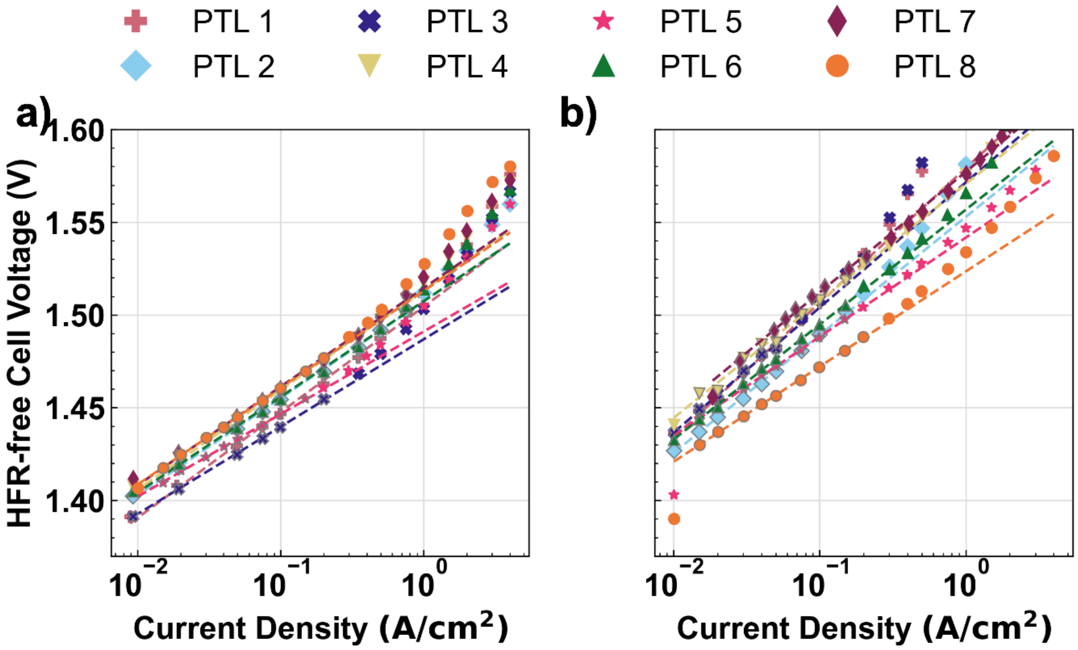

Figure S5 – Tafel plots (HFR-free voltage vs. log current density) for the a) 0.4 mg_{Ir} cm^{-2} and b) 0.1 mg_{Ir} cm^{-2} cells.

Figure S6:

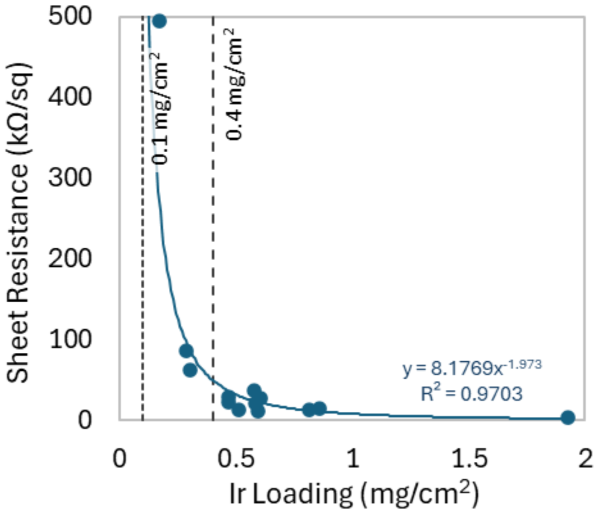

Figure S6 – Anode catalyst layer in-plane sheet resistance as a function of Ir loading at 80°C in dry N_2; the solid line depicts a power law fit which is included for visualization.

Figure S7:

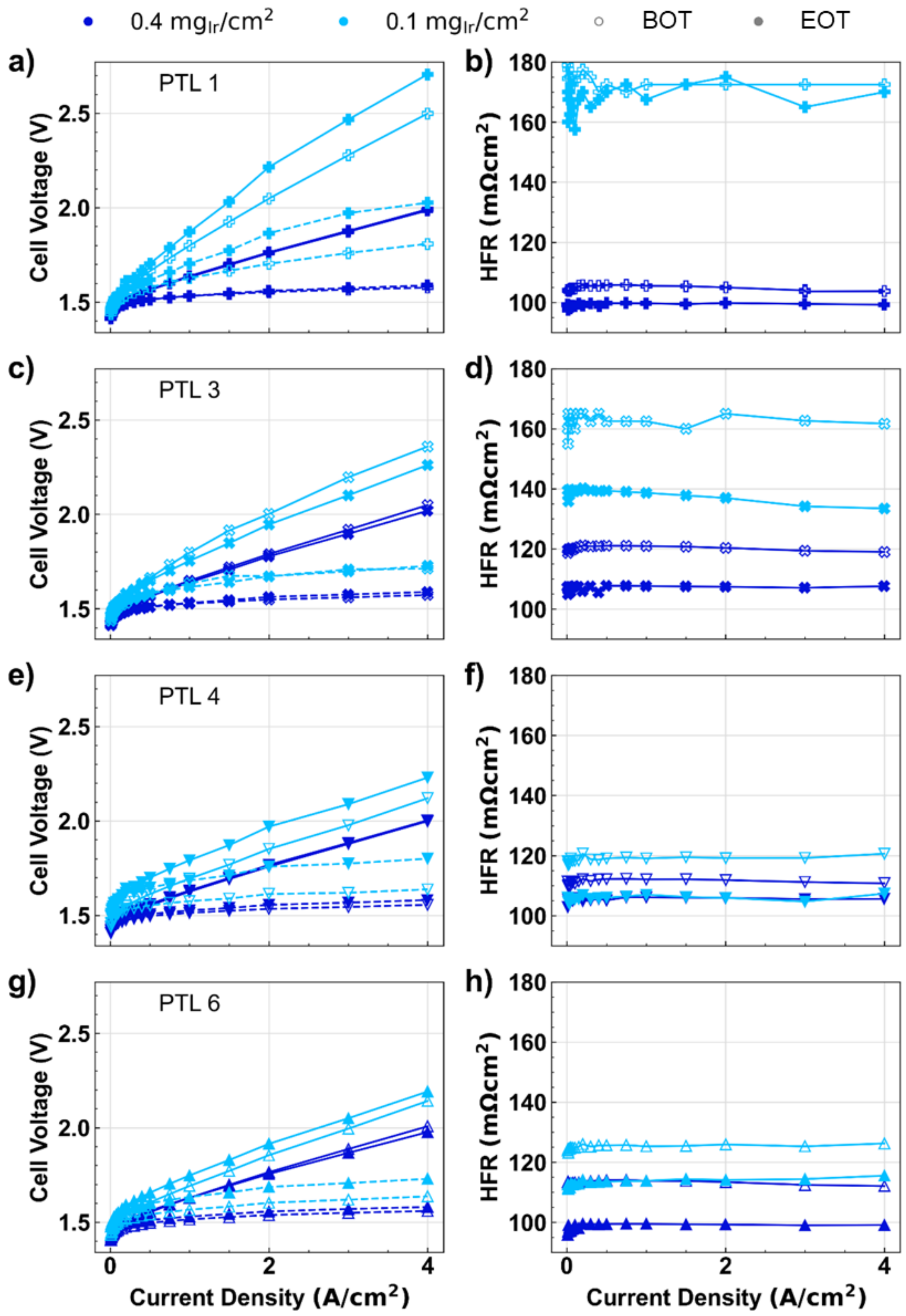

Figure S7 - BOT vs. EOT polarization curve comparison across PTLs and Ir loadings. The left column (a,c,e,g) shows cell voltages and IR-free voltages, and the right column (b,d,f,h) shows HFRs.

Figure S8:

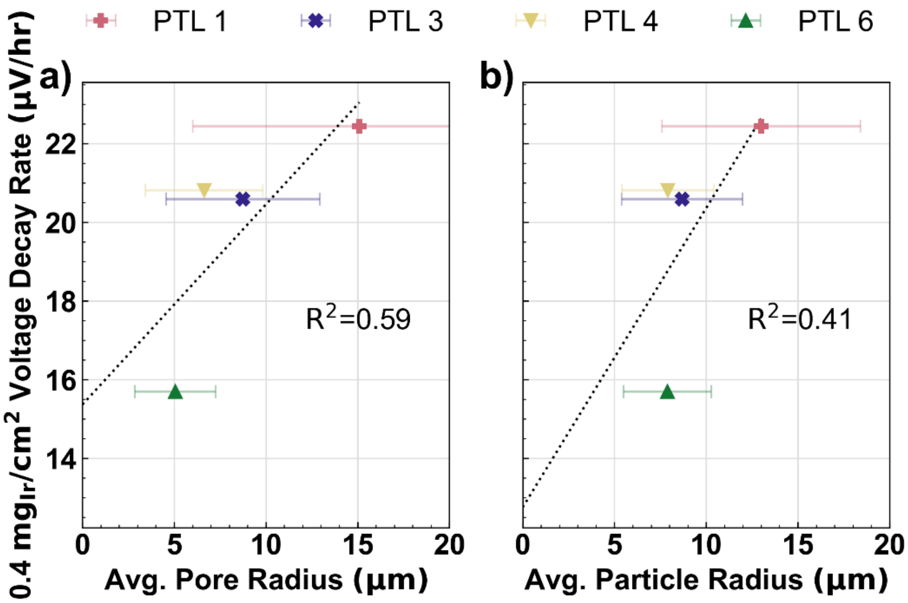

Figure S8 – Voltage decay rate of 0.4 mg$_{Ir}$ cm^{-2} samples during 1000-hour galvanostatic hold (2 A cm^{-2}) vs. a) average PTL pore radius, b) average PTL particle radius.

Figure S9:

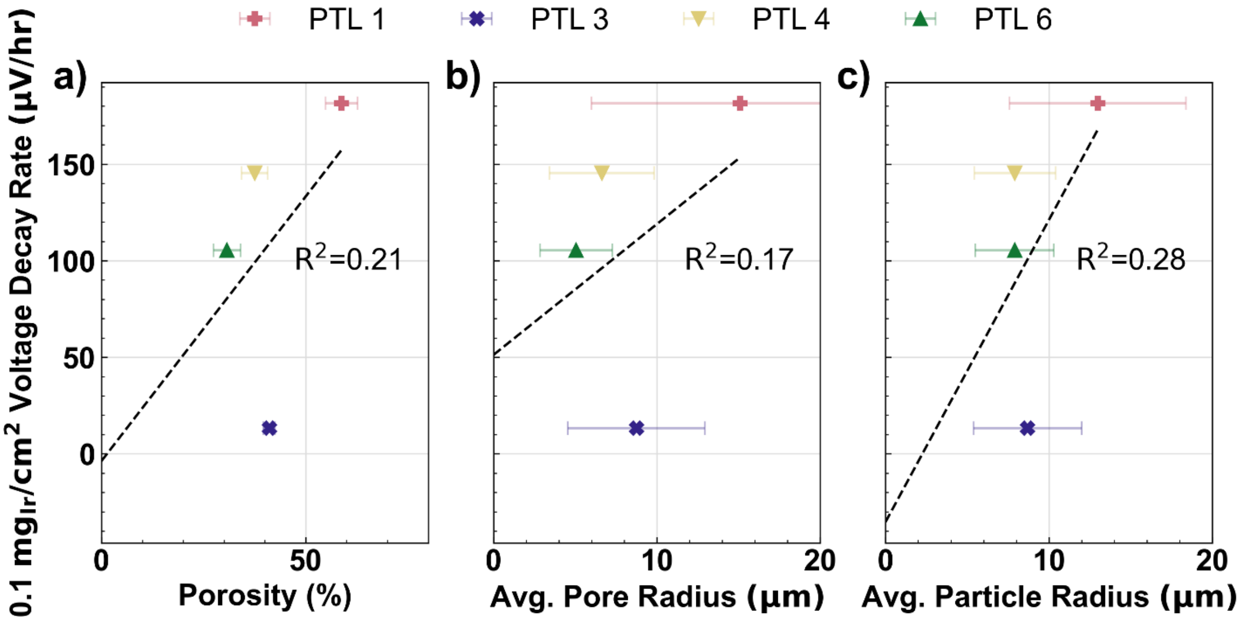

Figure S9 – Voltage decay rate of 0.1 mg$_{Ir}$ cm^{-2} samples during 1000-hour galvanostatic hold (2 A cm^{-2}) vs. PTL properties: a) porosity, b) average pore radius, and c) average particle radius.

Figure S10:

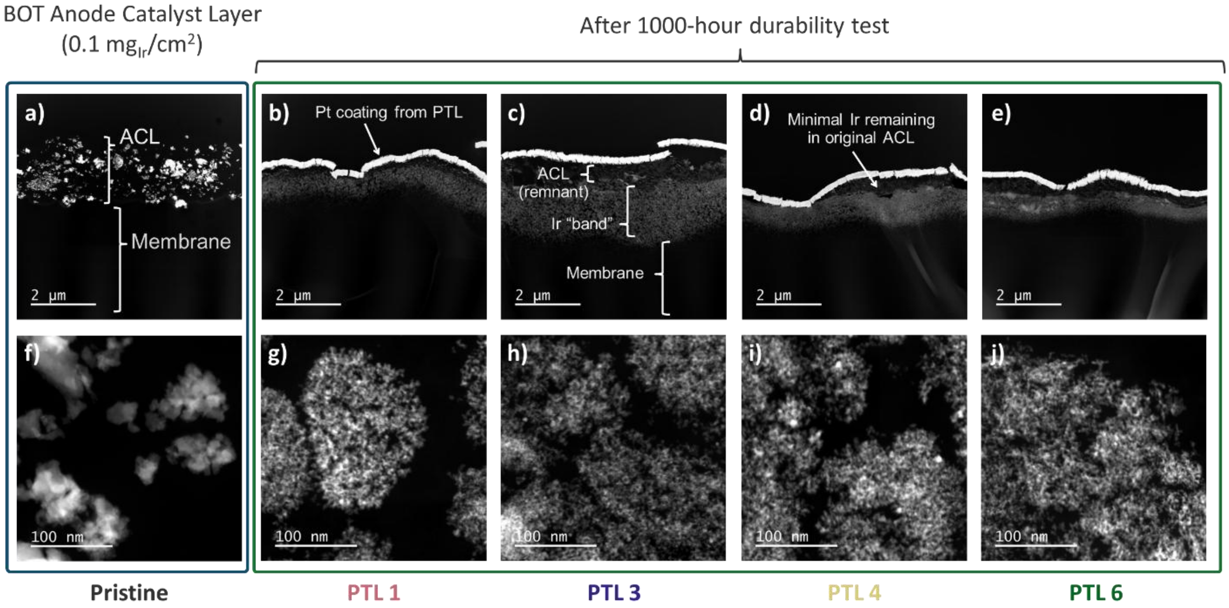

Figure S10 – HAADF-STEM images the 0.1 mg_{Ir} cm^{-2} anode catalyst layer cross sections before (a & f) and (b-e & g-j) 1000-hours of testing (each column is from a cell using a different PTL, top and bottom rows show two magnifications)

Figure S11:

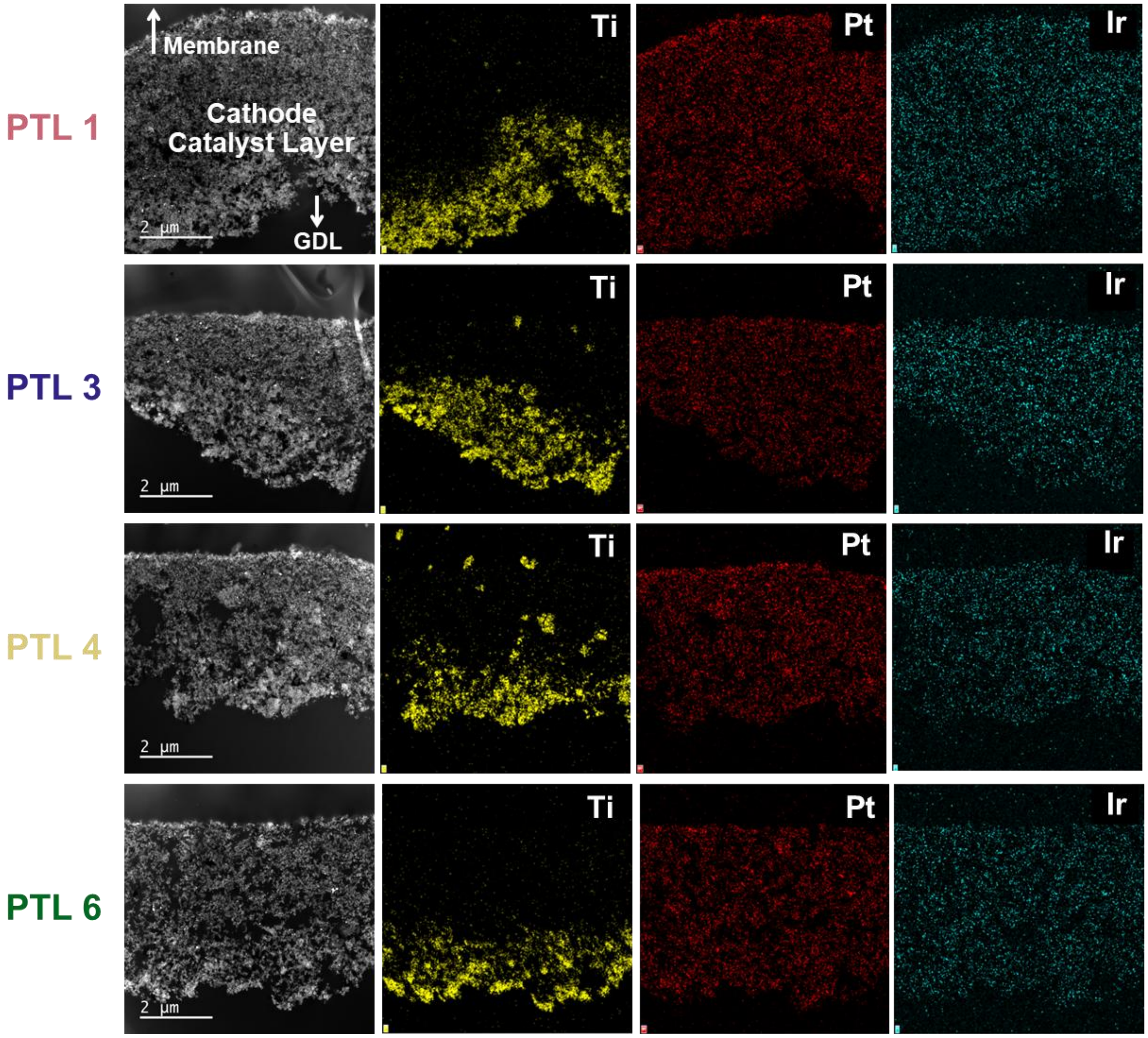

Figure S11 – STEM-EDS images of the cathode catalyst layer cross sections after 1000-hours of testing; the rows depicts images from cells using a particular PTL and the columns depict grayscale and elemental maps of Ti, Pt, and Ir.

Figure S12:

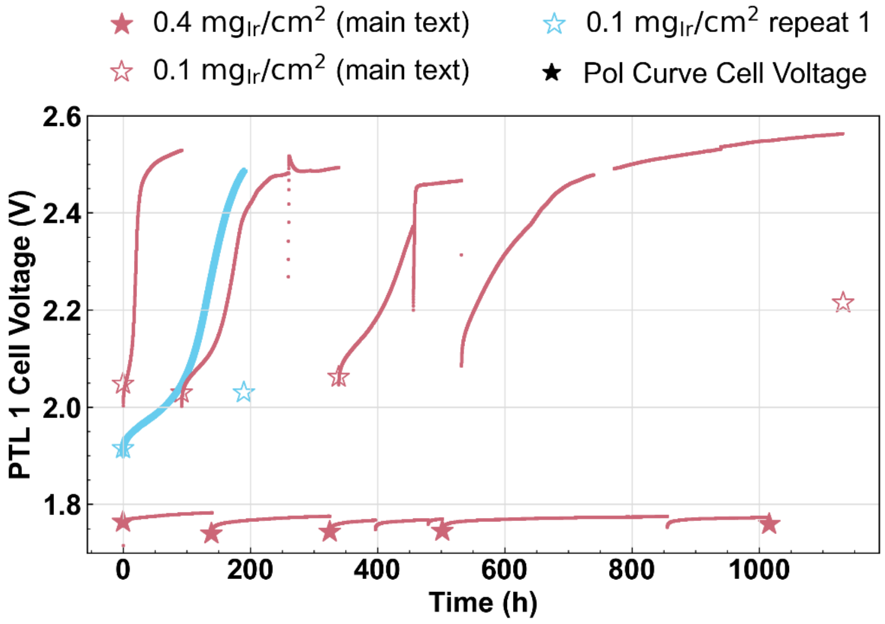

Figure S12 – Replicate durability test (blue) for PTL 1 using 0.1 mg_{Ir} cm^{-2} CCM (data from main text is replicated in magenta).

Figure S13:

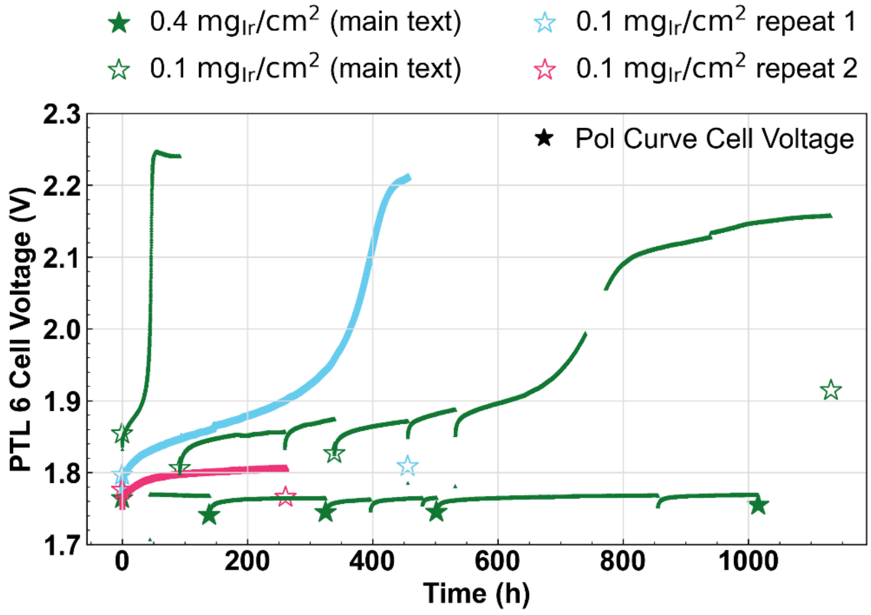

Figure S13 – Replicate durability tests (blue and magenta) for PTL 6 using 0.1 mg_{Ir} cm^{-2} CCMs (data from main text is replicated in green)
